# A Generalized Polymer Precursor Ink Design for 3D Printing of Functional Metal Oxides

**DOI:** 10.1007/s40820-023-01147-w

**Published:** 2023-07-13

**Authors:** Hehao Chen, Jizhe Wang, Siying Peng, Dongna Liu, Wei Yan, Xinggang Shang, Boyu Zhang, Yuan Yao, Yue Hui, Nanjia Zhou

**Affiliations:** 1 Key Laboratory of 3D Micro/Nano Fabrication and Characterization of Zhejiang Province, School of Engineering and Research Center for Industries of the Future, Westlake University, Hangzhou, 310030 People’s Republic of China; 2 Institute of Advanced Technology, Westlake Institute for Advanced Study, Hangzhou, 310024 People’s Republic of China; 3 School of Materials Science and Engineering, Zhejiang University, Hangzhou, 310027 People’s Republic of China; 4 School of Chemical Engineering and Advanced Materials, the University of Adelaide, Adelaide, 5005 Australia

**Keywords:** 3D printing, Maillard reaction, Polymer-assisted deposition, Metal oxide, Photonic crystal

## Abstract

Three-dimensional-structured metal oxides have myriad applications for optoelectronic devices. Comparing to conventional lithography-based manufacturing methods which face significant challenges for 3D device architectures, additive manufacturing approaches such as direct ink writing offer convenient, on-demand manufacturing of 3D oxides with high resolutions down to sub-micrometer scales. However, the lack of a universal ink design strategy greatly limits the choices of printable oxides. Here, a universal, facile synthetic strategy is developed for direct ink writable polymer precursor inks based on metal-polymer coordination effect. Specifically, polyethyleneimine functionalized by ethylenediaminetetraacetic acid is employed as the polymer matrix for adsorbing targeted metal ions. Next, glucose is introduced as a crosslinker for endowing the polymer precursor inks with a thermosetting property required for 3D printing via the Maillard reaction. For demonstrations, binary (i.e., ZnO, CuO, In_2_O_3_, Ga_2_O_3_, TiO_2_, and Y_2_O_3_) and ternary metal oxides (i.e., BaTiO_3_ and SrTiO_3_) are printed into 3D architectures with sub-micrometer resolution by extruding the inks through ultrafine nozzles. Upon thermal crosslinking and pyrolysis, the 3D microarchitectures with woodpile geometries exhibit strong light-matter coupling in the mid-infrared region. The design strategy for printable inks opens a new pathway toward 3D-printed optoelectronic devices based on functional oxides.

**Supplementary Information:**

The online version contains supplementary material available at 10.1007/s40820-023-01147-w.

## Highlights

- A facile and generalized design strategy of polymer precursor inks was developed for direct ink writing of metal oxide into submicron 3D architectures.
- The Maillard reaction between polyethyleneimine and glucose endows the 3D-printed precursors with the excellent shape fidelity during high-temperature pyrolysis.
- As-printed 3D periodic dielectric structure with woodpile geometry shows a significant light-matter effect in mid-infrared region.

**Supplementary Information:**

The online version contains supplementary material available at 10.1007/s40820-023-01147-w.

## Graphical abstract

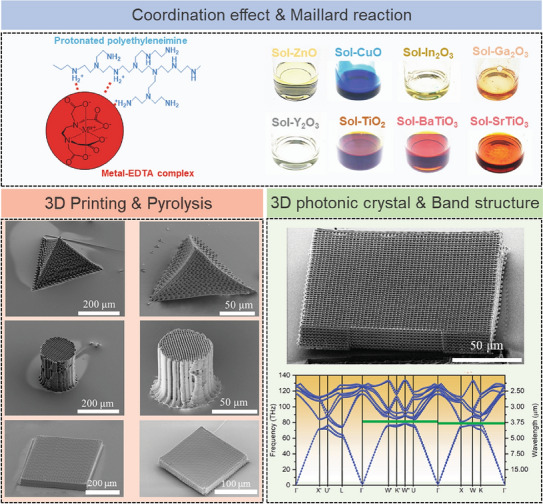

## Introduction

Metal oxides represent an important class of materials for the electronics industry [1, 2], owing to their diverse properties such as piezoelectricity [3], superconductivity [4], ferromagnetism [5] and electrochemical activity [6]. Patterning metal oxide into micro/nanometer-scale 2D and 3D architectures are significant not only for improving device performance [7–9], but also for driving new device architecture designs and proposing new heterogeneous integration strategies in the post-Moore era [10–12]. In particular, oxides with 3D architectures show interesting characteristics which are usually unachievable by those with planar structures [13], such as 3D photonic crystal with a full photonic bandgap [14], anisotropic electromechanical response generated through designed piezoelectric architectural units [15], damage-tolerant mechanical properties [16, 17] and higher mass transfer efficiency for gas sensing [18], photocatalytic [9, 19] and energy storage applications [20]. Conventional manufacturing strategies for 3D-structured metal oxides rely mostly on complex lithographic processes, which are capital intensive and could face significant challenges for constructing 3D oxide architectures [21, 22]. To meet the ever-increasing demand of 3D metal oxide devices, new cost-effective 3D manufacturing strategies and materials are highly desirable.

Recently, additive manufacturing technologies such as two-photon lithography (TPL) and ink-based printing strategies have started to demonstrate their potential for the rapid and high-resolution fabrication of on-demand nano/micro structured metal oxides. For example, TPL has been explored for the fabrication of various oxides from photopolymerizable polymer precursor materials consisting of targeted metal ligand compounds. However, most photoresins require complex composition design and optimization for each new polymer precursor to ensure the graft of photopolymerizable groups on metallo-organics [23–25] or form metal acrylates [14, 26–28]. Recently, Saccone et al. reported a hydrogel infusion-assisted additive manufacturing method for the fabrication of 3D-structured metal oxides and metals by first printing hydrogel scaffolds and subsequently infiltrating them with different metal ions [29]. Yet, the resolution of printed oxides is approximately 40 µm. Besides, this approach also faces challenges when printing oxides with hydrolytic metal ions such as titanium.

Instead of using photopolymerizable precursor materials, ink-based printing approaches directly deposit materials, and they offer greatly expanded choices of printable materials beyond photocurable ones. Among them, direct ink writing (DIW) represents a convenient platform for 3D-printed metal oxides down to sub-micrometer resolution. Due to the favorable rheological characteristics, DIW enables the controllable construction of 3D metal oxide structures with spanning features typically unachievable using inkjet printing [30, 31] or near-field electrospinning (NFES) [32, 33]. Upon printing, further sintering and calcination steps guarantee the structural fidelity and functionalities of the printed oxides [34]. The printable metal oxide inks are mainly categorized into two types, i.e., nanoparticle-based and polymer precursor-based inks. For nanoparticle-based ink formulation, despite that a diverse selection of commercially available oxide nanoparticles can be readily formulated into colloidal inks for DIW, the printing resolution using those inks are typically limited to several micrometer to hundreds of micrometers due to the size of particles and their agglomeration [18, 35]. For high-resolution printing ideally down to submicron resolution, particle-free polymer precursor inks based on sol–gel chemistry are highly desirable [36, 37]. Typically formed by hydrolysis and condensation of metallo-organics in organic solvent, these polymer precursor inks consist of soluble linear polymer chains with homogeneous phase characteristics at the molecular level, and they are ideal for extrusion through ultrafine nozzles with inner diameter as small as ~ 1 μm [36]. Despite these promises, polymer precursor inks for 3D-printed oxides are extremely rare mainly because most metallo-organic ligands with high chemical reactivity readily form highly branched agglomerates which eventually crosslink to form non-printable colloidal gels upon solvent evaporation [38].

**Fig. 1:**
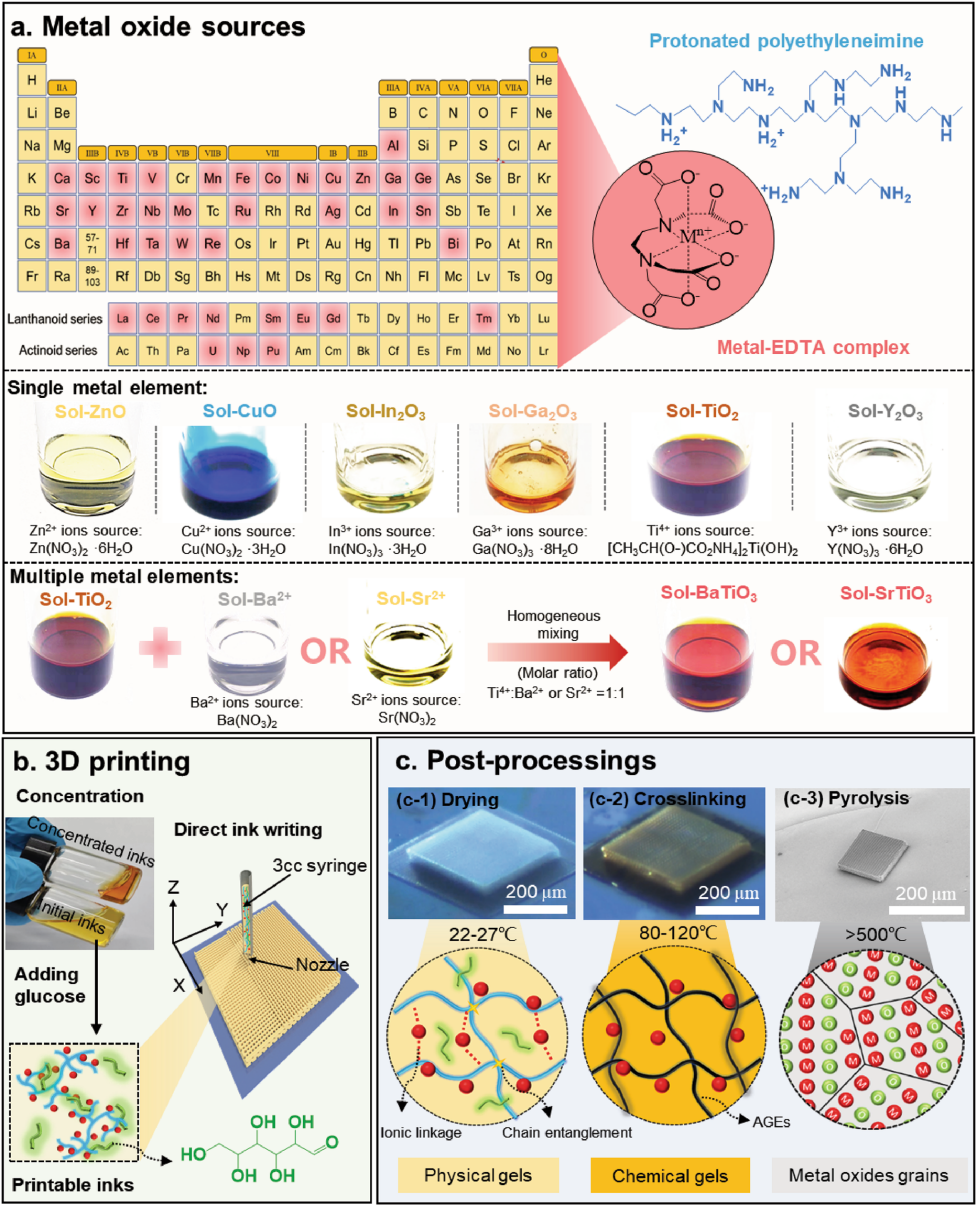
Universal design of 3D printable polymer precursor inks and their post-processing steps toward 3D metal oxides: **a** Chemical structure of protonated PEI binding metal-EDTA complex. M^n+^ represent metal ions capable of forming stable complexes (the light red in the element periodic table [40, 44]). Among these, we demonstrate the preparation of polymer precursor inks including a single metal element (e.g., Zn^2+^, Cu^2+^, In^3+^, Ga^3+^, Ti^4+^, and Y^3+^) and multiple metal elements (e.g., Ba^2+^ and Ti^4+^, Sr^2+^ and Ti^4+^). **b** Initial inks were concentrated by evaporating the excess solvent and then homogenized after dissolving a certain amount of glucose as crosslinker to form printable inks. **c** 3D-printed precursor inks can be completely converted into targeted metal oxides with 3D structures in a series of post-processing steps: **c-1** drying, **c-2** thermal crosslinking, and subsequent **c-3** pyrolysis in air. (Color figure online)

**Fig. 2:**
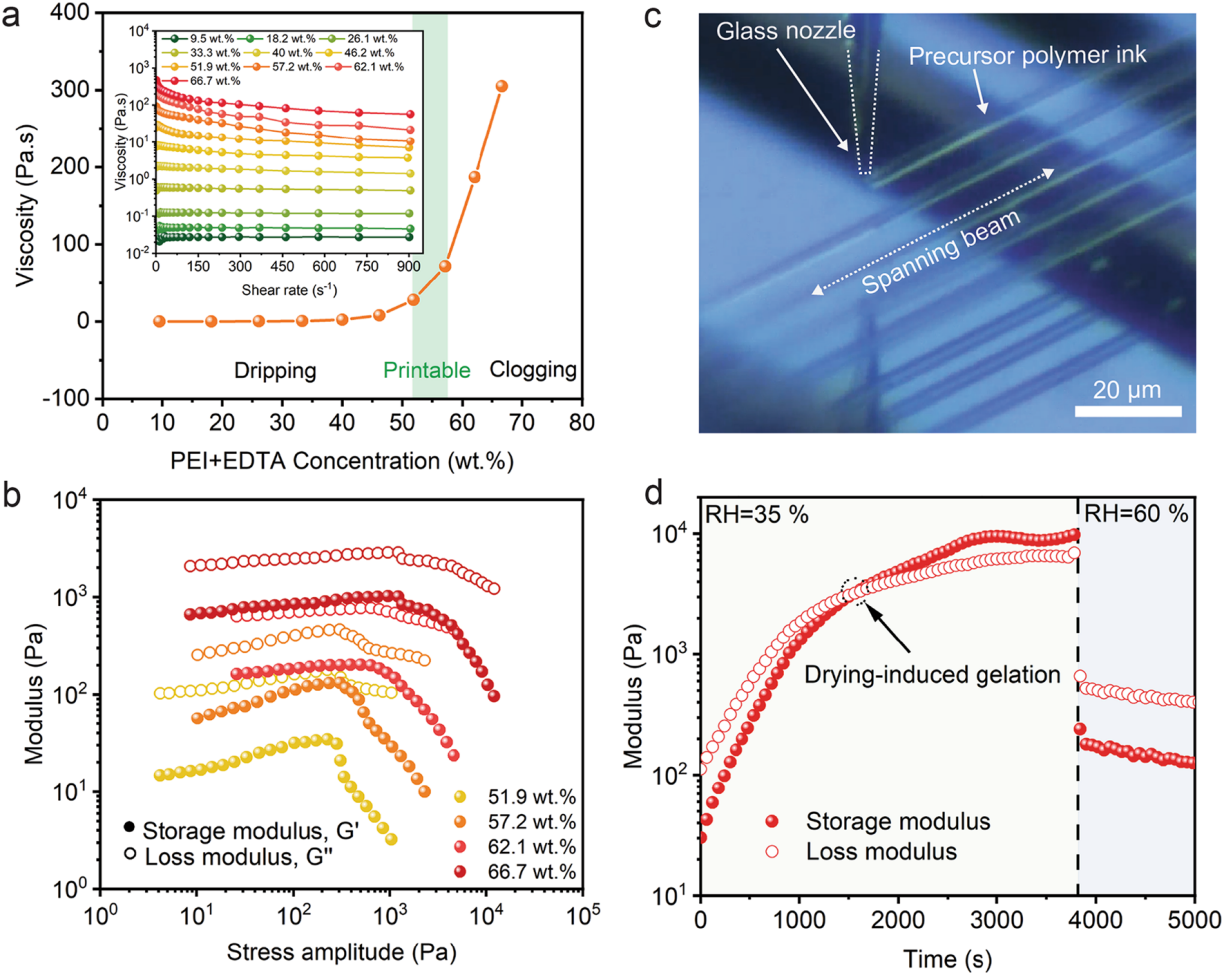
Rheological properties of 3D printable polymer inks: **a** Ink viscosity at a shear rate of 100 s^−1^ as a function of total concentration of PEI and EDTA (PEI: EDTA = 1:1, by weight). Inset shows viscosity as a function of shear rate for polymer inks of varying PEI/EDTA concentration. **b** Storage and loss moduli as a function of shear amplitude for polymer inks of varying PEI/EDTA concentration. **c** Optical image of inks extruded through a 2 µm glass nozzle as spanning filaments on a substrate composed of parallel rectangular silicon ribbons. **d** Storage and loss moduli as a function of time for inks with 51.9 wt% of PEI/EDTA concentration upon exposure to different humidity environment (oscillatory frequency = 1 Hz, stress amplitude = 10 Pa). The drying-induced gelation occurs in the moment of ink extrusion and endows PEI/EDTA inks with shape-retaining property

**Fig. 3:**
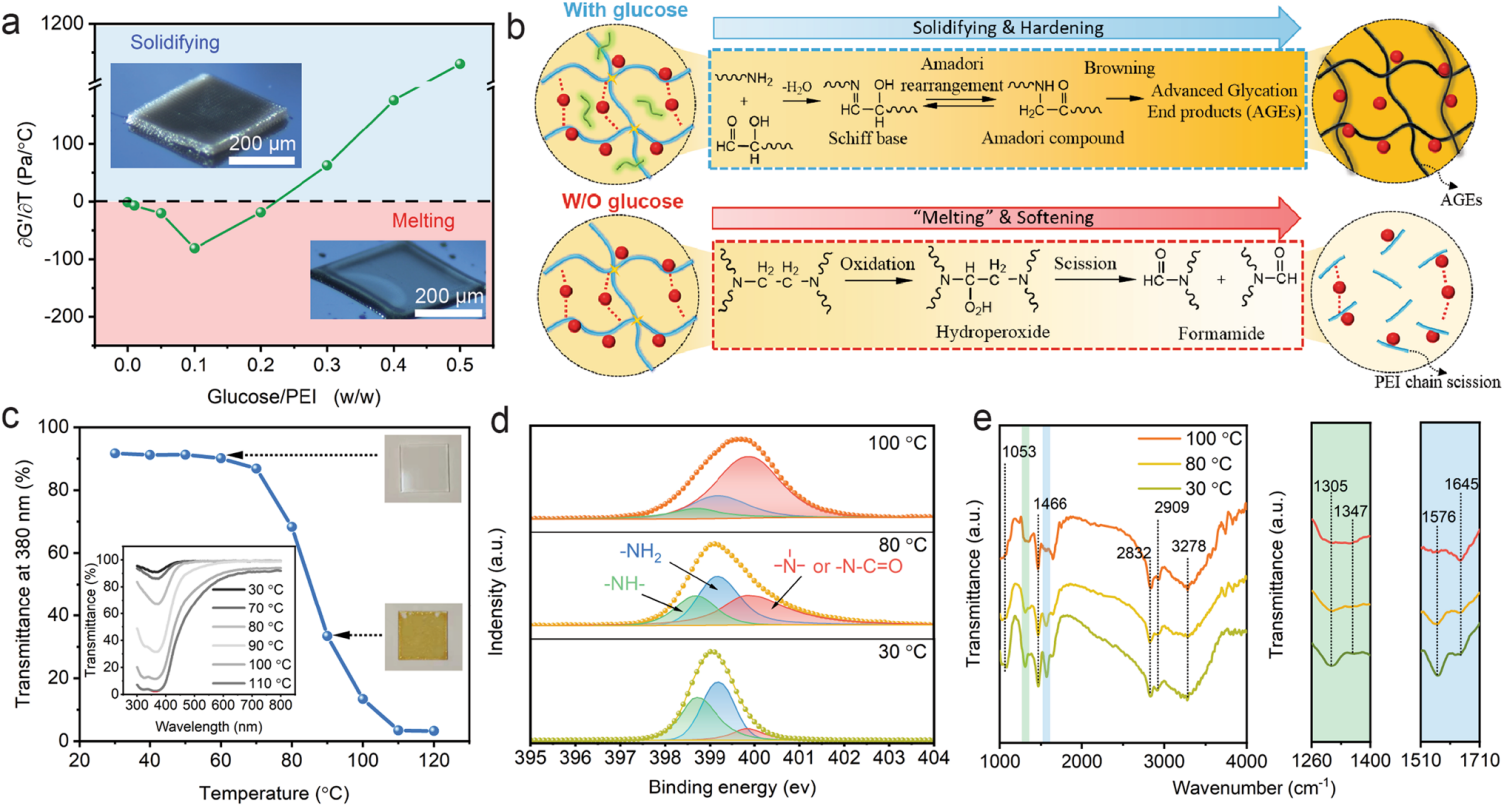
Thermal crosslinking mechanisms of the polymer precursors with glucose:** a** Derivative of the storage modulus with respect to temperature at ~ 105 °C as a function of glucose-to-PEI weight ratio. Inset images show the morphologies of 3D polymer precursors after heating to 200 °C with adequate (upper left) and inadequate (bottom right) glucose contents. **b** Illustration highlights two chemical reaction routes possibly in thermal crosslinking of polymer inks. The blue arrow indicates that polyethyleneimine (PEI) undergoes Maillard reaction with glucose to form a dark brown and robust resin, i.e., advanced glycation end products (AGEs). The red arrow reveals the “melting” and softening phenomenon possibly attributed to chain scission during PEI degradation. **c** Transmittance change of 380 nm light with temperature increasing from 30 to 120 °C. Inset shows the UV–vis transmittance spectra for precursor films on glass substrates after heating to different temperatures for 1 h. **d** N 1*s* XPS spectra of PEI/glucose mixture after heating to 30, 80 and 100 °C for 1 h. The dotted lines are the raw data. After deconvolution using Gauss–Lorentz curves, the blue, green, and red curves represent primary, secondary, and tertiary amino or amide groups centered at 399.16, 398.63, and 399.83 eV, respectively. **e** In situ ATR-FTIR spectra obtained from the same freeze-dried PEI/glucose mixture samples, heated at 30, 80, and 100 °C for 5 min. (Color figure online)

In this work, we developed a generalized ink formulation strategy for the DIW of functional metal oxides 3D architectures with a submicron printing resolution by taking advantages of the coordination of metal ions with polyethyleneimine (PEI) functionalized by ethylenediaminetetraacetic acid (EDTA). This coordination chemistry could be generally employed for formulating a wide range of metal–organic complexes while preventing them from hydrolysis (Fig. 1a), thereby ensuring a stable and homogeneous composition in aqueous solution after ultrafiltration [39, 40]. We investigated the optimal rheological properties required for ultrafine resolution 3D printing by regulating polymer solution concentration upon solvent evaporation (Fig. 1b). The printed precursor polymer exhibited an excellent self-supportive characteristic due to PEI polymer chain entanglement and electrostatic interaction between PEI and EDTA in the physical gels (Fig. 1c, c-1). To prevent the collapse of printed structures during pyrolysis, glucose was introduced to thermally crosslink with the polymer matrix (PEI) to form chemical gels by Maillard reaction (Fig. 1c, c-2). For our demonstration, we printed various metal ion-containing polymer precursor inks with nozzle diameter as small as ~ 2 µm. 3D-printed binary (i.e., ZnO, CuO_,_ In_2_O_3_, Ga_2_O_3_, TiO_2_, and Y_2_O_3_) and tertiary metal oxides (BaTiO_3_ and SrTiO_3_) were obtained via organic-to-inorganic transformation through high-temperature pyrolysis. The observed dramatic shrinkage of the 3D structures can be attributed to the thermal degradation of organics (Fig. 1c, c-3). Finally, we demonstrate 3D-printed ZnO structures with the face-centered tetragonal geometry showing a pseudo-photonic bandgap (PBG) centered at 3.47–3.72 µm which is consistent with plane wave expansion simulations.

## Experimental Section

### Materials

Polyethyleneimine solution (PEI solution, average M_n_ ~ 60,000 by GPC, 50 wt% in H_2_O), zinc nitrate hexahydrate (Zn (NO_3_)_2_·6H_2_O, 98%) and titanium (IV) bis (ammonium lacato) dihydroxide solution (TALH solution, 50 wt% in H_2_O), gallium nitrate octahydrate (Ga(NO_3_)_3_·8H_2_O, 99.9%) were purchased from Sigma-Aldrich. copper nitrate trihydrate (Cu(NO_3_)_2_·3H_2_O, 99%) was purchased from Adamas-beta. yttrium nitrate hexahydrate (Y(NO_3_)_3_·6H_2_O, 99.9%), indium nitrate (In(NO_3_)_3_·4H_2_O, 99.9%), barium nitrate (Ba(NO_3_)_2_, 99%), strontium nitrate (Sr(NO_3_)_2_, 99%), ethylene diamine tetraacetic acid (EDTA, 99.9%) and anhydrous D-glucose (99%) were purchased from Macklin Inc. Crystal bond™ 509 was purchased from Structure Probe, Inc.

### Synthesis of Binary Oxide Polymer Precursor Inks

A batch of EDTA/PEI polymer solutions were firstly prepared by dissolving 0.4 g of EDTA and 0.8 g of PEI solution into 25 mL of deionized water. For zinc ions-containing solution, 0.4 g of Zn(NO_3_)_2_·6H_2_O was dissolved into the polymer solution and stirred for 4 h, until the solution became transparent and light yellow. Subsequently, each metal ions-containing solution was diluted to 200 mL and then filtered through a 0.45 μm syringe filter. The filtered solution was placed in an Amicon stirred cell containing an ultrafiltration disk (Millipore Co., USA) designed to the retention of materials with a molecular weight of > 10 kDa. After ultrafiltration, the solution was concentrated to 20 mL in volume. By evaporating solvent, the polymer solution was further concentrated for the optimum rheological properties. Finally, 0.2 g of D-glucose was dissolved completely in the concentrated polymer inks by using a Thinky mixer (ARE-300, Thinky Co., Ltd., Tokyo, Japan).

The synthesis procedure of other metal ion-containing solution was similar to that of Zinc ion-containing solution, but replacing the Zn(NO_3_)_2_·6H_2_O by the corresponding metal salts, i.e., 0.33 g of Cu(NO_3_)_2_·3H_2_O, 0.51 g of In(NO_3_)_3_·3H_2_O, 0.55 g of Ga(NO_3_)_3_·8H_2_O, and 0.52 g of Y(NO_3_)_3_·6H_2_O and 0.805 g of TALH solution.

### Synthesis of Ternary Oxide Polymer Precursor Ink

For BaTiO_3_ polymer precursor ink, barium ions-containing solution were prepared by firstly dissolving 0.4 g of EDTA and 0.8 g of PEI solution into 25 mL of deionized water. Then, 0.358 g of Ba(NO_3_)_2_ was added to this solution and stirred for 4 h until the solution became transparent and colorless. The barium ions-containing solution was diluted to 200 mL and then filtered through a 0.45 µm syringe filter. The filtered solution was concentrated to 20 mL after ultrafiltration process. The titanium and barium ions-containing solutions were mixed to yield a solution with equal molar concentration of titanium and barium. The mixed solution was further concentrated by evaporating solvent and 0.4 g of D-glucose was dissolved completely in the concentrated polymer ink using a Thinky mixer. For SrTiO_3_ polymer precursor ink, 0.358 g of Ba(NO_3_)_2_ was replaced by 0.29 g of Sr(NO_3_)_2_, and subsequent steps were similar.

### Ink Rheology

The rheological properties of the polymer precursor solution were investigated using a controlled stress rheometer (Discovery HR 10, TA Instrument Co., Ltd., US) with a 25 mm-diameter plane plates, and the rheological samples were loaded in the 200-µm gap between parallel planes. For each ink with a certain concentration of PEI and EDTA (by weight), its viscosity was measured as a function of shear rate from 0.1 to 1000 s^−1^. The viscosity measured at an approximate shear rate of 100 s^−1^ was reported as a function of total concentration of PEI and EDTA. In oscillation measurement, the storage and loss moduli of the inks with varying concentrations were measured by stress amplitude sweep at a frequency of 1 Hz. The drying-induced gelling processes were estimated by a real-time scanning mode, where the applied stress amplitude was fixed at 10 Pa for collecting moduli data of the printable ink (51.9 wt%) in a dry (relative humidity of 35%) and a wet (relative humidity of 60%) environment. The temperature-dependent moduli were investigated for the printable ink using temperature scanning mode at a fixed stress amplitude of 10 Pa, with a temperature ramp from 25 to 150 °C at 5 °C min^−1^.

### Ink Uniformity

The metal ions-containing solutions with and without EDTA were prepared and then diluted 100,000-fold in volume. The hydrodynamic diameter distribution for each solution was characterized by wide-angle dynamic light scattering analysis (Brookhaven Inc., US). All measurements were taken three times for 1 min each at 25 °C. Additionally, sedimentation tests were performed on tenfold diluted solution.

### Spectroscopic Characterization for Maillard Reaction

For UV–vis spectrum measurement, PEI/glucose (2:1 by weight) solutions were firstly coated on the quartz substrate and then heated to different temperature for 10 min. After that they were placed in the solid-state sample holder, while the reference sample holder was a clean quartz substrate. The transmittance spectra from 300 to 800 nm of the samples were measured using a UV spectrometer (UV 2700, Shimadzu Co., Japan) and the transmittance data measured at 380 nm were reported as a function of heating temperature. X-ray photoelectron (XPS) spectroscopy analysis of the PEI/glucose (2:1 by weight) mixtures coated on the silicon wafer after heating to 30, 80 and 100 °C for 10 min was conducted on the ESCALAB Xi + spectrometer (Thermo Fisher Scientific Ltd., Germany). For Fourier transform infrared (FTIR) characterization, the PEI/glucose (2:1 by weight) solutions were firstly freeze-dried to form a gel-like solid samples and then placed on the sample holder of attenuated total reflectance (ATR) accessory. The sample was immersed for 10 min at 30, 80 and 100 °C in sequence, before the corresponding FTIR spectrum were acquired on an In situ ATR-FTIR spectrometer (IN 10, Thermo Fisher Scientific Ltd., Germany) in a range from 1000 to 4000 cm^−1^.

### Adsorption Behavior of Zn^2+^ with PEI/EDTA Mixture and PEI Polymer under Different pH Environment

A batch of PEI/EDTA-Zn^2+^ solutions was prepared by mixing 0.4 g of EDTA, 0.8 g of PEI solution (50 wt% in water) and 0.4 g of Zn (NO_3_)_2_·6H_2_O and diluted to 200 mL with distilled water. The pH values of these solutions were, respectively, tailored as 2, 4, 6, 8, 10 and 11 by adding a suitable amount of HNO_3_ and NH_3_·H_2_O. After stirring for 24 h, these solutions concentrated to 50 mL in volume by ultrafiltration process. Subsequently, the concentrated solutions were diluted to 10,000 times its original weight with 1 wt% HNO_3_, while the Zn^2+^ ions concentration in the diluted solution was lower than 176 ppb (by weight). The diluted solutions were then centrifuged at 6000 rpm for 10 min and the solutions sampled in the upper layer were analyzed for the Zn^2+^ ions concentration with inductively coupled plasma mass spectrometry (ICP-MS, iCAP RQ, Thermo Fisher Scientific Ltd., Germany). For adsorption analysis of Zn^2+^ ions with PEI polymer, the PEI-Zn^2+^ solutions were firstly prepared by mixing 0.8 g of PEI solution and 0.4 g of Zn (NO_3_)_2_·6H_2_O. Likewise, the subsequent processes of sample preparation and characterization were described as the above.

### Saturation Adsorption Capacity of Zn^2+^ in PEI/EDTA Solution

PEI/EDTA aqueous solutions were firstly prepared by dissolving 0.4 g of EDTA into 200 g of PEI solution (0.2 wt% in water). Then, 0.2, 0.3, 0.4, 0.5, 0.6, 0.7 and 0.8 mg of Zn (NO_3_)_2_·6H_2_O were added into the PEI/EDTA solutions, respectively. The pH values of these solutions were fixed at 4 by adding a suitable amount of HNO_3_. The subsequent procedures for sample preparation and characterization are the same as mentioned above. The weight ratio of Zn^2+^ ions to PEI was reported as a function of the molar ratio of Zn^2+^ ions to EDTA.

### Ink Characterization for Organic-to-Inorganic Transformation

The mass loss of polymer precursor ink was performed on thermogravimetry and differential scanning calorimetry (TGA/DSC 3+/1600 HT, Mettler-Toledo Co., Ltd, Switzerland) from 50 to 800 °C at a heating rate of 10 °C min^−1^ in air. X-ray diffraction (XRD) patterns of pyrolyzed products at different temperatures were collected by a powder X-ray diffractometer (D8 Advance, Bruker Co., Ltd., Germany). The organic elemental compositions (C, H, O, and N) were directly measured using an element analyzer (Unicube, Elementar, Germany), while the metal-ions concentration was determined by subtracting the total organic elemental contents.

#### 3D Printing of Metal Oxide Structure

Direct ink writing (DIW) was performed using a custom-built dual-drive air-bearing 3-axis microposition platform, whose motion pathway is commanded by G-code. The polymer precursor inks were placed on a 3-cc syringe barrel connected to 2-µm glass micronozzle produced using a P-2000 micropipette puller (Sutter Instrument Co., Novato, CA, USA). The syringe barrel was installed in a high-pressure dispensing tool (HP3cc, EFD Inc., East Providence, RI, USA) actuated by an air-powered fluid dispenser (Performus™ V, EFD Inc., East Providence, RI, USA). The required printing parameters for patterning metal oxides were 20–30 psi of air pressure and 200–600 µm s^−1^ of printing speed.

For 1D and 2D planar pattern, the polymer precursor can be directly deposited on a silicon wafer. The spanning structure was deposited on a substrate composed of parallel rectangular silicon microribbons. This substrate was fabricated by lithography and etching processing using a Semi-Automated Mask Aligner (MA/BA6 Gen4, Suss Micro Tec., Lithography Inc., Germany). In particular, the 3D-structured polymer precursor was printed onto a sacrificial organic layer which was deposited on double-polished silicon wafers. The sacrificial organic solution was prepared by dissolving Crystal bond™ 509 in acetone (15 wt% in acetone) and then coated on the silicon wafer at 2000 rpm for 30 s using a spinning coater (KW-4A, Chemat Techology Inc., USA), after the wafer was modified by plasma surface treatment (PCE-6, Kejing Materials Technology Co. Ltd., Hefei, China). The sacrificial layer melts above 121 °C, allowing the release of 3D structures from the silicon wafer. This results in uniform shrinkage of 3D structures without any constraint from the substrate during the pyrolysis process. All 3D printing processes and subsequent crosslinking reaction (100 °C for 8 h) were carried out in ambient environment with a relative humidity of 35–40%. The printed structures were pyrolyzed in air in a muffle furnace (KSL-1200X-J, Kejing Materials Technology Co. Ltd., Hefei, China). For 3D-structured ZnO, the temperature was ramped up to 650 °C at 1 °C min^−1^ and maintained at 650 °C for 1 h. For 3D-structured CuO, In_2_O_3_, TiO_2_, Y_2_O_3_, and SrTiO_3_, the temperature was ramped up to 600 °C at 1 °C min^−1^ and maintained at 600 °C for 1 h. For 3D-structured BaTiO_3_, the temperature was ramped up to 800 °C at 1 °C min^−1^ and maintained at 800 °C for 1 h. For 3D-structured Ga_2_O_3_, the temperature was ramped up to 700 °C at 1 °C min^−1^ and maintained at 700 °C for 1 h.

#### Characterization of 3D-Printed Structure

The printed structure (before and after pyrolysis) was observed by scanning electron microscope (Gemini 450, Carl Zeiss Co., Ltd, Germany) and element distribution was performed by energy-dispersive X-ray spectrometry detectors (Extreme, Oxford Instrument, UK). Transmission electron microscopy (TEM) images and diffraction patterns were obtained using a high-resolution transmission electron microscope (Talos F200X G2, Thermo Fisher Scientific Ltd., Germany). Statistical distribution of grain size was obtained by manually measuring the diameter of 100 particles with the ImageJ software. Cross-sectional images of the 3D-structured ZnO with woodpile geometry are obtained using focused ion beam milling (Gemini SEM Crossbeam 550, Zeiss, Germany).

#### Optical Measurement and Simulations

Reflectance and transmittance spectra of 3D ZnO woodpile were acquired using a Fourier transform infrared (FTIR) microspectrometer (Nicolet iS50, Thermo Fisher Scientific Ltd., Germany) equipped with a Cassegrain objective. The angle range of incident light was between 16° and 35° relative to the normal. The reflectance spectra were normalized to a gold mirror and the transmittance spectra were normalized to double-sided polished silicon wafer that served as a substrate for the 3D ZnO woodpile. All the simulations were calculated by the commercial software COMSOL Multiphysics. The required structural parameters for the simulations were measured from the printed 3D woodpiles. The photonic band structure was analyzed in the first Brillouin zone by an eigenfrequency solver in the frequency domain module. For simulated transmittance spectra and electric field magnitude, the continuous periodic boundary conditions were applied in four planes perpendicular to x and y directions, while an excitation port and an output port were set on the top and bottom planes, respectively. S and P-polarized plane waves were considered in the excitation port, and its propagation direction was determined by the azimuth (*θ*) and elevation (*φ*) angles, as shown in Fig. S22.

## Results and Discussion

### Universal Design of 3D Printable Polymer Precursor Inks

To synthesize the metal ion-containing precursor inks, we first prepare an aqueous solution by mixing EDTA and PEI with a 1:1 weight ratio and subsequently add targeted metal element sources. Almost all metal ions are coordinated into stable metal-EDTA complexes with negative charges. As a typical cationic polyelectrolyte, PEI polymer could offer high-density positive charge distribution, resulting in strong electrostatic attraction between protonated PEI and metal-EDTA complexes (Fig. 1a). The membrane ultrafiltration was adapted for the removal of unwanted molecules and unbound metal ions, effectively preventing the metal salts and hydroxides from precipitation during concentration. The chemical stability of the resulting ink is significant during solvent evaporation. Here, we estimate that the optimal PEI/EDTA concentration of polymer inks and metal ions-containing inks, as highlighted by the shaded region in Figs. 2a and S1. By contrast, the inks with lower PEI/EDTA concentrations show stronger capillary effect, resulting in the formation of droplets at the nozzle tips (Fig. S2, Dripping). Upon increasing the concentration of PEI/EDTA, we observed that the viscosity of the ink displays shear-thinning property at higher shear rates [41–43]. On the other hand, the more concentrated inks result nozzle clogging due to the solvent evaporation (Fig. S2, Clogging). The storage moduli (*G*′) are always lower than loss moduli (*G″*) with an increasing PEI/EDTA concentration (Fig. 2b), implying that the inks exhibit a liquid-like response and are not able to form self-supportive spanning features required for 3D printing (Fig. 2c). To further understand the ink solidification mechanisms, oscillatory rheology measurements are conducted for 51.9 wt% ink (Fig. 2d). As solvent evaporates in dry environment (relative humidity, RH ~ 35%), the inks gradually exhibit a solid-like behavior (*G′* *>* *G″*). When exposed to humid air (RH ~ 60%), the inks returned to show a liquid-like behavior (*G′* < *G″*). This humidity-dependent viscoelastic response is ideal for DIW, allowing rapid ink solidification upon printing. This gelation process is dominated by the chain entanglement of PEI polymers and the electrostatic attraction between EDTA and PEI (Fig. 1c, c-1). Notably, the printed microscale filaments have a much higher specific surface area than the bulk material, resulting in an accelerated solvent loss of the printed filaments. In fact, the printability tests reveal that the physical gelation of the printed filaments could occur in an environment with a relative humidity of 35 ~ 40%.

### Thermal Crosslinking Mechanism

The structural integrity of the printed samples upon pyrolysis depends strongly on the thermosetting behaviors of the polymer precursors which effectively prevent the 3D structures from softening or collapsing during the organic-to-inorganic transformation [45]. For the precursor inks without glucose, with an increasing temperature, their storage moduli move closer to their loss moduli. However, an abrupt drop in both storage and loss moduli occurs at ~ 105 °C (Fig. S3a), corresponding to the collapsing and spreading of the 3D-printed structures in a process similar to melting (Fig. 3a, bottom right). One possible reason for this phenomenon is the chain scission during thermal degradation of PEI, which involves the activation of carbon–carbon bond by an initially formed hydroperoxide and then breaks into two formamide groups (Fig. 3b, bottom) [46]. The resulting polymers with shorter chains exhibit lower storage moduli than loss moduli which fail to withstand structure fidelity at ~ 105 °C. To overcome such abrupt phase change in printed 3D structures, high-density and permanent covalent cross-links are required. Thanks to the presence of amino groups in PEI polymers, we conduct the Maillard reaction for crosslinking PEI polymers by adding a certain amount of glucose. Many reports in food chemistry have demonstrated that Maillard reaction is a sophisticated polymerization reaction with three main stages [47] (Fig. 3b, top): (i) condensation, (ii) Amadori rearrangement, (iii) browning. Among them, the known mechanism of the initial stage in Maillard reaction involves the condensation between an amino group of a nitrogenous compound and a carbonyl group of reducing sugar. The rheological measurements show that the addition of glucose to precursor inks leads to the crossover point of storage and loss moduli occurring at ~ 95 °C, indicating the formation of crosslinked networks (Fig. S3b-h). However, when glucose/PEI ratios are less than 0.3, a significant drop in these inks moduli still occurs above 105 °C (Fig. S3b–e). Increasing the glucose-to-PEI weight ratio to 0.3 ensures that the derivative of the storage modulus with respect to temperatures remains positive at all temperature points (Fig. 3a). As the temperature increases from 95 to 110 °C, the storage modulus increases by three orders of magnitude continuously (Fig. S3f–h), and the 3D-structured polymer precursor still exhibits the excellent shape-retaining ability (Fig. 3a, upper left). It implies that sufficient crosslinking density were required for preventing the structure collapse due to polymer chain scission.

Meanwhile, we next employed a suite of spectroscopic techniques to characterize the Maillard reaction in PEI/glucose mixtures. The color of PEI/glucose film changes from colorless to dark brown as a proof of the nonenzymatic browning processing (Fig. 3c), which is an important characteristic of Maillard reaction [48, 49]. These resulting dark brown polymeric aggregates known as advanced glycation end products (AGEs) are mainly formed from further crosslinking between Amadori compounds and adjacent amino groups and they exhibit thermosetting property (Fig. 1c, c-2) [47]. We next investigated the changes in the functional groups on PEI after thermal crosslinking by X-ray photoelectron spectroscopy (Fig. 3d). N1s signals of all samples can be deconvoluted into three peaks at the binding energies of 399.16, 398.63 and 399.83 eV, corresponding to primary, secondary, and tertiary amino groups or amide groups, respectively [50–53]. The shift to higher binding energy of the original N1s peak implies that a large number of primary and secondary amino groups are converted into tertiary amino and amide groups during the Maillard reaction. We also obtained FT-IR spectra on the PEI/glucose films which are heated to 30, 80 and 100 °C using an attenuated total reflectance (ATR) module (Fig. 3e and Table S1). A broadening of peak in the range of 1260–1400 cm^−1^ (green) is a result of the increasing number of –CH groups (1347 cm^−1^), possibly attributed to the graft of glucose chains on PEI polymer. Meanwhile, the disappearance of the peak at 1576 cm^−1^ (blue) demonstrates that –NH groups participate in the reaction with glucose. Notably, the characteristic peaks of –NH_2_ and –C=N groups overlap with each other. Since –NH_2_ groups are consumed to form tertiary amino and amide groups, the increase in the intensity of the peak at 1654 cm^−1^ suggests the appearance of Schiff base (–C=N groups).

### DIW of Metal Oxides

**Fig. 4:**
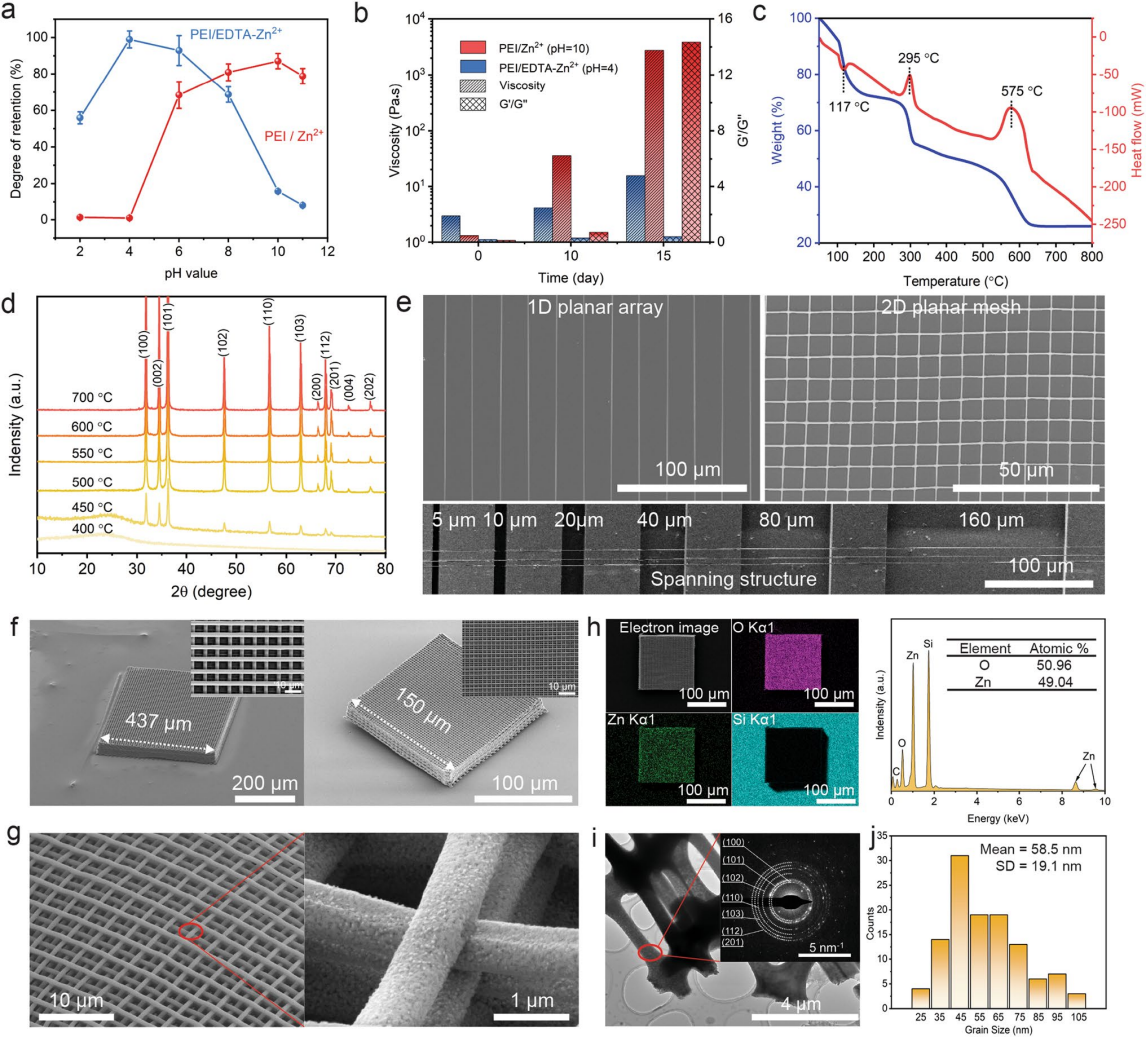
3D printing of ZnO architectures: **a** The adsorbed Zn^2+^ ions content of purified precursor inks (blue: PEI/EDTA-Zn^2+^, red: PEI/Zn^2+^) with varying pH values, measured by ICP-MS analysis. **b** The effect of pH value on long-term rheological stability demonstrated by the viscosity (shear rate = 1 s^−1^) and the ratio of *G*′ to *G*″ (in the linear viscoelastic range) of inks stored in ambient environment for 0, 10 and 15 days. **c** Thermogravimetry and differential scanning calorimetry (TG-DSC) curves of zinc-containing ink. **d** X-ray patterns obtained from samples of crosslinked zinc-containing inks sintered at different temperatures ranging from 400 to 700 °C for 1 h. **e** SEM images of spanning structure, 1D array, and 2D mesh (patterned with a 2-µm nozzle) after sintering the zinc-containing ink at 650 °C for 1 h. **f** A 32-layers polymer precursor woodpile (left) printed with a 2-µm nozzle was converted to the ZnO structure (right) upon sintering at 650 °C for 1 h. **g** High-magnification surface morphologies of 3D-structured ZnO. **h** Element distribution and the corresponding EDS spectrum of as-fabricated ZnO woodpile. **i** Low-magnification TEM image of printed ZnO structure and an electron diffraction pattern (inset). **j** Grain size distribution of 100 particles measured from the dark and bright field TEM images (Fig. S11). (Color figure online)

For the design of direct ink writable metal oxide polymer precursor inks, sufficient targeted metal ions should be coordinated in the crosslinked polymer networks to avoid fragmentation of the printed structures after pyrolysis [36]. Therefore, we evaluate the adsorption effect of polymers on metal ions for maximizing the amount of zinc ions in polymer precursor inks after ultrafiltration (Fig. 4a). In an alkaline environment, only non-protonated PEI polymers can produce the lone-pair electrons on the nitrogen atoms and directly coordinate with certain metal cations, such as Zn^2+^, Cu^2+^, Ba^2+^, and Sr^2+^ for the formation of homogeneous and transparent complex solutions (Fig. S4a, b, g, h). However, the alkaline environment not only causes the highly reactive metal ions (such as In^3+^, Ga^3+^, Ti^4+^, and Y^3+^) to hydrolyze and precipitate (Fig. S4c–f), but also triggers the nucleophilic attack by non-protonated amino group on the carbonyl group of the glucose to form chemically crosslinked gels even at room temperature [54], resulting in significantly increased ink viscosities and moduli (Figs. 4b and S5). These problems can be solved by introducing EDTA to chelate with metal ions. Different from PEI/Zn^2+^, the metal-EDTA complexes with negative charges are bound to the protonated PEI electrostatically (Figs. 1a and S4). The maximum retention of Zn^2+^ ions appears in a pH 4 environment where both amine groups of PEI polymers and partial carboxyl groups of EDTA are ionized [55, 56]. At room temperature, the rheological properties of PEI/EDTA-Zn^2+^ precursor ink remain stable in a long period of 15 days (Fig. 4b). Based on this composition design in pH 4 solution, the optimal molar ratio of Zn^2+^ to EDTA is determined as 1:1 for achieving the maximal adsorption of PEI to Zn^2+^ after ultrafiltration (Fig. S6). Besides, the cross-sectional morphologies shows that the saturated adsorption of PEI to Zn^2+^ endows extruded ink filaments with rod-like structures after pyrolysis at 650 °C (Fig. S7). The organic-to-inorganic transformation for the polymer precursor is strongly dependent on pyrolysis temperature, which undergoes three typical stages as indicated by TGA–DSC profiles (Fig. 4c). First, the solvent evaporation results in an endothermic peak at 117 °C. Next, a rapid weight loss occurs along with an exothermic reaction at ~ 295 °C which is due to the degradation of oxygen-containing groups (Fig. S8). Finally, as the temperature increases further to 630 °C, the organic compositions are eliminated in a relatively long exothermic process. X-ray diffraction patterns reveal that the pyrolyzed products are identified as ZnO phase with an onset crystallization temperature of ~ 450 °C (Fig. 4d).

To demonstrate the high-resolution printing of metal oxide structures achievable with this method, we printed 1D planar array and 2D planar mesh composed of ZnO filaments by extruding polymer precursor inks through a capillary nozzle with a 2 µm inner diameter (Fig. 4e, top). Upon pyrolysis at 700 °C, polymer decomposition results in a reduction of filament width down to ~ 550 nm while the grain growth induces the appearance of nanopores at the grain interface (Fig. S9). Interestingly, the spanning structures of ZnO filaments printed on Si microribbons still maintain their structural fidelity upon pyrolysis (Fig. 4e, bottom). We next printed complex 3D architectures, such as 150-layer cylinder, 100-layer pyramid and 32-layer woodpile, which are composed of parallel arrays of orthogonally stacked filaments (Figs. S10 and 4f). The first layer of the structure is printed on substrates pre-coated with a sacrificial organic layer to mitigate anisotropic shrinkage of the underlying structure. During the pyrolysis process at 650 °C, the 32-layer woodpile undergoes isotropic shrinkage with the overall lateral dimensions change from 437 to 150 µm (Fig. 4f), yet it still retains its initial geometry with final microfilament diameter of 766 nm (Fig. 4g). The energy-dispersive X-ray spectroscopy (EDS) elemental maps of the pyrolyzed structures reveal a uniform distribution of zinc, oxygen, and carbon in the whole structure without apparent element segregation (Fig. 4h, left), showing atomic percentages of Zn and O of 49.04 and 50.96%, respectively (Fig. 4h, right), which is consistent with that of ZnO powders from elemental analysis (Fig. S8). To confirm the conversion of 3D-printed polymer precursor to monolithic polycrystalline ZnO, we analyze the atomic-level microstructures of the 3D-printed structures by transmission electron microscopy (TEM). The electron diffraction pattern taken from the red region confirm the hexagonal wurtzite crystalline phase of ZnO (Fig. 4i), while the bright and dark field TEM images reveal that the pyrolyzed ZnO structures is composed of nanocrystalline with a mean grain size of 58.5 nm (Figs. 4j and S11). Besides ZnO, our ink formulation strategy can be readily applied to a variety of metal ions, such as Cu^2+^ ions for CuO, In^3+^ ions for In_2_O_3_, Ga^3+^ ions for Ga_2_O_3_, Ti^4+^ ions for TiO_2_, and Y^3+^ ions for Y_2_O_3_ (Figs. S12-S16). We also demonstrate the 3D printing of some ternary metal oxides, such as SrTiO_3_ and BaTiO_3_ (Figs. S17–S18).

### Optical Properties of 3D-Printed ZnO Woodpile Architectures

**Fig. 5:**
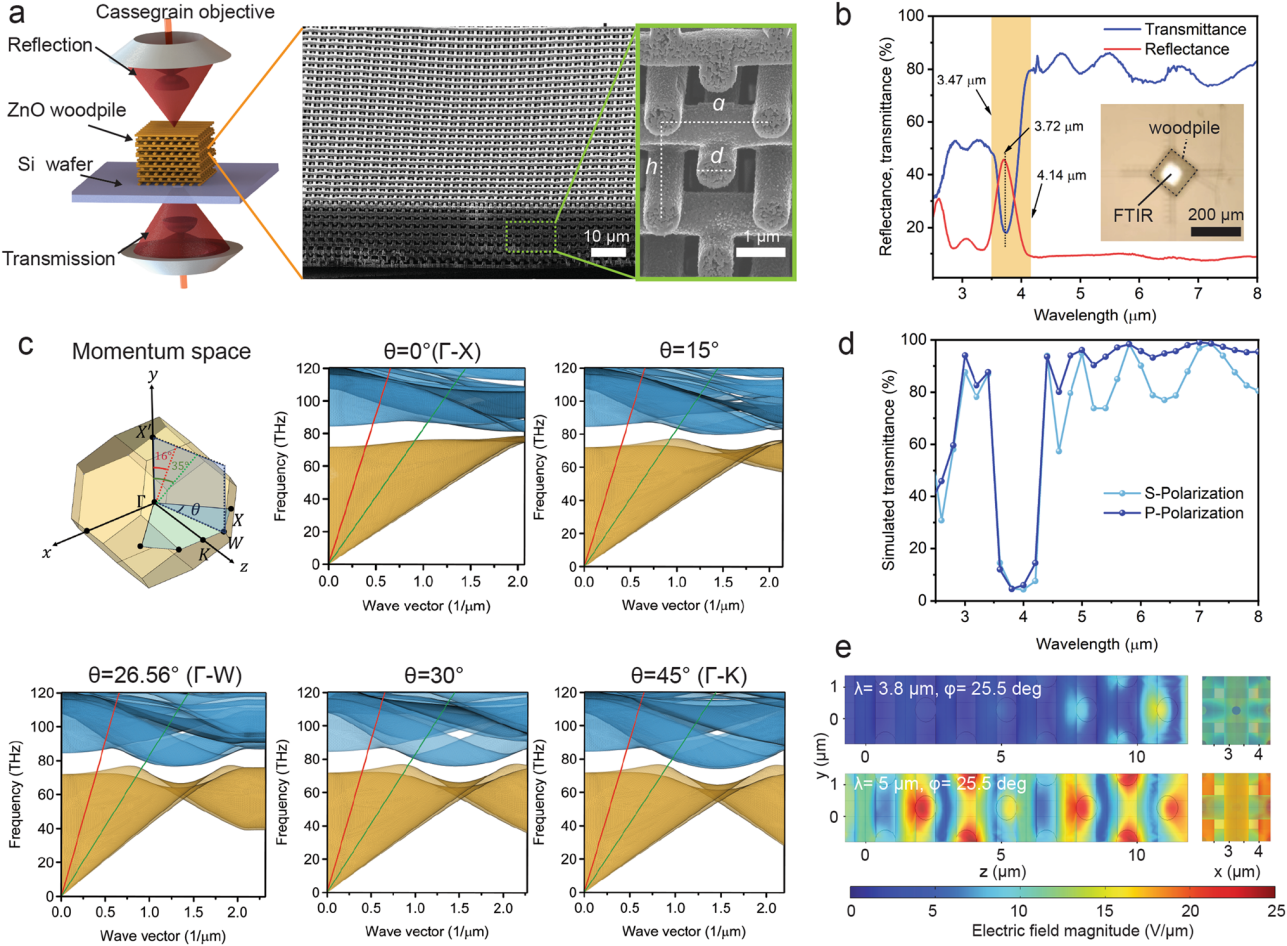
Optical characterization and simulation analysis of ZnO woodpile: **a** Illustration of a standard FT-IR measurement setup with a Cassegrain objective having an acceptance angle range between 16° and 35°, and SEM images of focused ion beam (FIB)-milled cross section of the woodpile structure, obtained with a substrate tilt angle of 55°. **b** FTIR reflectance and transmittance spectra taken from the ZnO woodpile, and the calculated bandgap in orange region. **c** Illustration for the first Brillouin zone of the FCT Bravais lattice and calculated band structures projected along ГX′ orientation, with varying azimuth angle, *θ*. **d** Simulated transmittance spectra of S and P-polarized light for 16-layer woodpile structure. **e** Calculated electric field distributions along the *xy* and *yz* planes, as the P-polarized light propagates with an elevation angle (*φ*) of 25.5° (an averaged acceptance angle of Cassegrain objective) and an azimuth angle (*θ*) of 0°. (Color figure online)

We next demonstrate 3D-printed ZnO woodpiles as a three-dimensional photonic crystal. The cross-sectional SEM images of the woodpile samples reveal that the average diameter ($d$) of the individual filament is ~ 0.766 µm and the average in-plane distance ($a$) between the two adjacent filaments is ~ 2.151 µm in every layer (Fig. 5a). Each layer rotates 90° relative to the layer below and offsets by $\frac{1}{2}a$ from two layers below. Hence, the structure repeats itself every four layers in the stacking direction, and the four-layer distance ($h$) is calculated as 2.961 µm, taking into account tilt correction for the cross-sectional SEM measurement. Notably, the ZnO woodpile belongs to a face-centered-tetragonal (FCT) Bravais lattice because the ratio of $h$ to $a$ is not strictly equal to $\sqrt{2}$. Given the geometric parameters of FCT lattice (Fig. S19a) and a refractive index of Zinc oxide (Fig. S20), we calculated the photonic band structure by probing along all symmetry points in the Brillouin zone of FCT lattice (Fig. S19b, Table S2). The calculated band diagram shows that existence of partial photonic bandgap (PBG) along the symmetry directions of Г–W′-K′–W″–U–Г and Г–X–W–K–Г at the wavelengths of 3.623–3.772 µm and 3.727–3.885 µm, respectively, while along symmetry direction of Г–X′–U′–L–Г, light propagation inside the photonic crystal was not prohibited in the above-mentioned wavelength ranges (Fig. S19c). It is because that the refractive index of ZnO is lower than the threshold value of ~ 2.3 required to open a complete PBG in FCT woodpile (Fig. S21). Nevertheless, a high reflectance/low transmittance band centered at 3.72 µm is detected using a Cassegrain objective with infrared light with an incident angle from 16° to 35° (Fig. 5a, b). To verify the experimental results, we map out the band structure projected along ГX′ orientation (Fig. 5c). The azimuth angle *θ* is set in range from 0° to 45°, considering the rotational symmetry of tetradecahedron geometry (the first Brillouin zone) about the *z*-axis. The red and green light lines are plotted in each projected band structure, corresponding to incidence angles of light at 16° and 35°, respectively. Each projected band structure predicts two sets of stop-band positions influenced by off-normal light incidences (Table S3). The group of all sets of stop-band positions indicates the existence of a partial PBG located between 4.14 and 3.47 µm, which match well with the peak and valley of the experimentally characterized reflectance and transmittance spectra (Fig. 5b). We next implement plane wave excitation to simulate the transmittance spectra for the 16-layer woodpile. To mimic actual angular spread of the incident light, we calculated spectra for several incident angle ($16^\circ \le \theta \le 35^\circ ,0^\circ \le \varphi \le 90^\circ$) in the respective relevant range and averaged over the obtained results associated with S and P- polarized light illumination (Figs. 5d and S22). The simulated and experimental spectra agree well on the edge of the partial bandgap. However, the deviations of transmittance depth are observed especially in the short wavelength region and partial PBG, attributed to the imperfect periodicity, surface roughness and uncertainty of refractive index [14, 57]. To further investigate the light propagation characteristics in the woodpile, two different electric field distribution profiles are calculated with incident light wavelengths of 5 and 3.8 µm (Fig. 5e). With 5 µm incident light, the electrical field was distributed over the entire simulated domain. By contrast, the significant attenuation of electric field amplitude occurs in the light propagation with the wavelengths of 3.8 µm, due to complete reflection. These results manifest the 3D-printed woodpile photonic crystals show strong coupling effects with mid-infrared light.

## Conclusions

In summary, we have developed a facile and generalized design strategy of polymer precursor inks for DIW of metal oxide into 3D architectures with a sub-micrometer resolution. Benefitting from the ink composition with strong adsorption effect on a wide range of metal ions, the design strategy has an ability to expand to other metal elements, and even potential to be compatible with multiple metal ions coexisted in aqueous solution. Furthermore, the homogeneous distribution of compositions at the molecular level facilitates ink extrusion continuously through a micronozzle. The polymer chain entanglement and electrostatic attraction effect are tailored easily upon the solvent evaporation resulting in the ideal rheological properties required for printability. The Maillard reaction between PEI and glucose endows the as-printed polymer precursor with the excellent shape-retaining property during the pyrolysis process. As a demonstration, we fabricated 2D- and 3D-structured metal oxides after the organic-to-inorganic transformation. We further demonstrated that as-printed 3D periodic dielectric structure with woodpile geometry has a significant light-matter effect in mid-infrared region. Importantly, our polymer precursor inks suggest a feasible and versatile pathway to the design and development of new functional oxide inks for DIW.

### Supplementary Information

Below is the link to the electronic supplementary material.

Supplementary file1 (PDF 2783 KB)
