# Supplementary material for: A Generalized Polymer Precursor Ink Design for 3D Printing of Functional Metal Oxides
Source: Nanomicro Lett. 2023 Jul 13;15:180. doi: 10.1007/s40820-023-01147-w (PMC10344857; doi:10.1007/s40820-023-01147-w)
Supplement: Supplementary file 1 — Supplementary file1 (PDF 2783 KB) [file 40820_2023_1147_MOESM1_ESM.pdf]

Supporting Information for

A Generalized Polymer Precursor Ink Design for 3D Printing of Functional Metal Oxides

Hehao Chen[1, 2, 3], Jizhe Wang[1, 2, 3], Siying Peng[1, 2], Dongna Liu[1, 2, 3], Wei Yan[1, 2], Xinggang Shang[1, 2], Boyu Zhang[1, 2], Yuan Yao[1, 2], Yue Hui[4], Nanjia Zhou[1, 2, *]

[1]Key laboratory of 3D Micro/Nano Fabrication and Characterization of Zhejiang Province, School of Engineering and Research Center for Industries of the Future, Westlake University, Hangzhou, 310030, P. R. China

[2]Institute of Advanced Technology, Westlake Institute for Advanced Study, Hangzhou, 310024, P. R. China

[3]School of Materials Science and Engineering, Zhejiang University, Hangzhou, 310027, P. R. China

[4]School of Chemical Engineering and Advanced Materials, the University of Adelaide, Adelaide, 5005, Australia

*Corresponding author. E-mail: zhounanjia@westlake.edu.cn (Nanjia Zhou)

Supplementary Figures and Tables

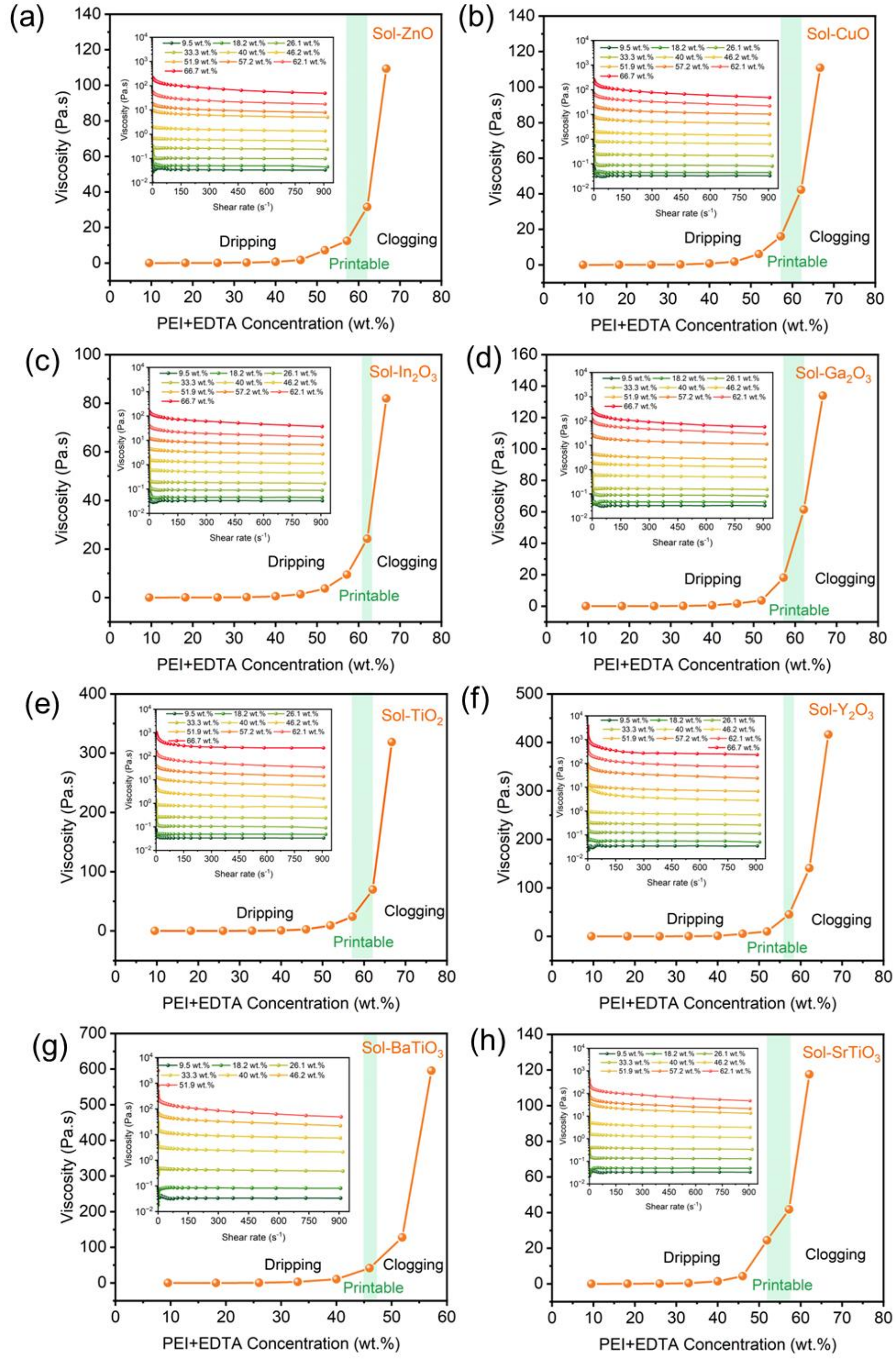

Fig. S1 Ink viscosities at a shear rate of 100 s^{-1} as a function of total concentration of PEI and EDTA (PEI: EDTA=1:1, by weight) for different metal ions-containing precursor inks, i.e., (**a**) Zn^{2+}, (**b**) Cu^{2+}, (**c**) In^{3+}, (**d**) Ga^{3+}, (**e**) Ti^{4+}, (**f**) Y^{3+} (**g**) Ba^{2+} and Ti^{4+}(1:1 molar ratio), (**h**) Sr^{2+} and Ti^{4+}(1:1 molar ratio). Inset shows the viscosity change as a function of shear rate

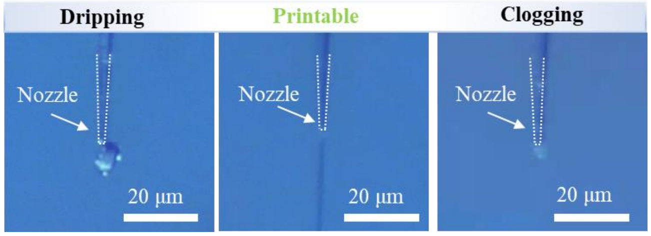

Fig. S2 Optical images capturing the process of inks with different PEI/EDTA concentrations extruding from the nozzle tips

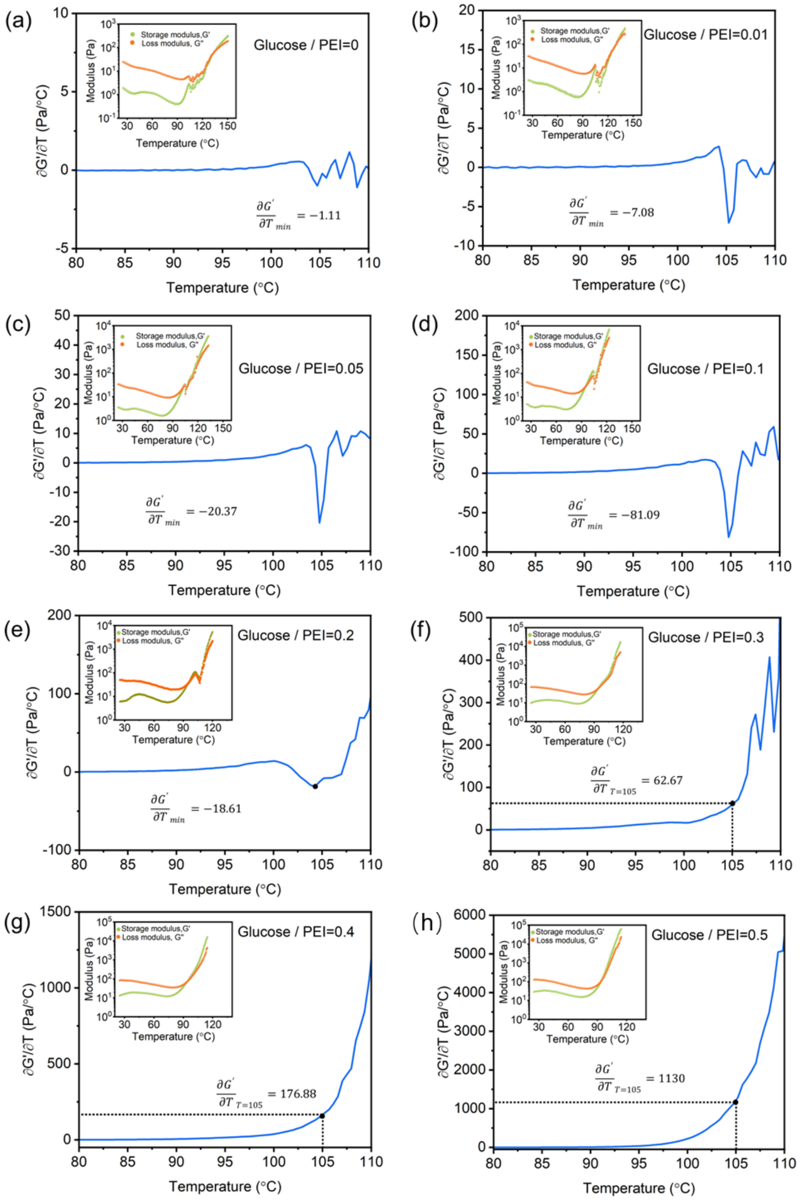

Fig. S3 The temperature-dependent derivative of the storage modulus as a function of temperature for precursor inks with varying glucose/PEI weight ratios, i.e., (**a**) 0, (**b**) 0.01,

(**c**) 0.05, (**d**) 0.1, (**e**) 0.2, (**f**) 0.3, (**g**) 0.4, (**h**) 0.5. Inset: the storage and loss moduli as a function of temperature

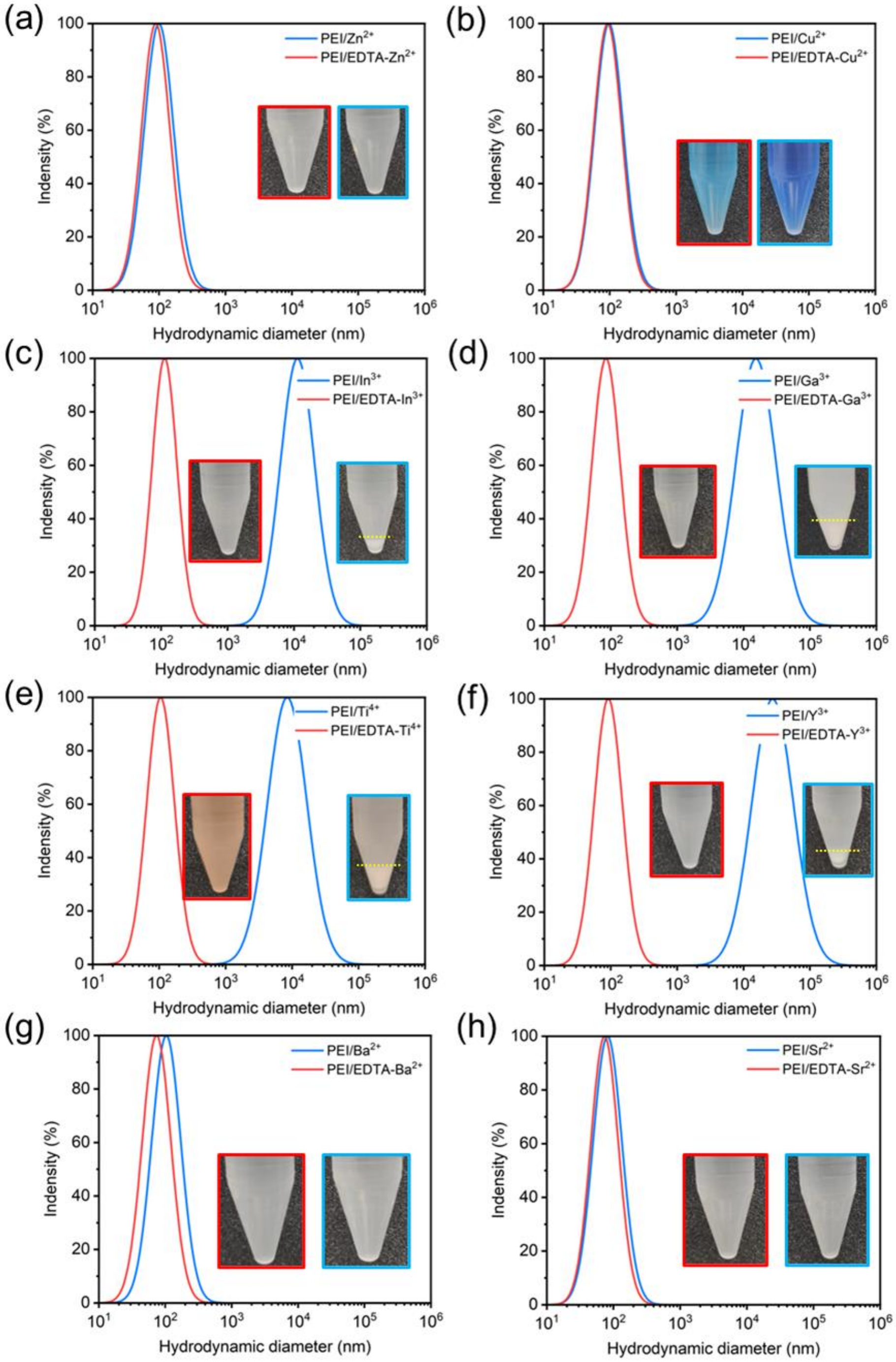

Fig. S4 The dynamic light scattering (DLS) analysis shows Lognormal size distributions of hydrodynamic diameters of PEI/metal ion (in blue) and PEI/EDTA-metal ion complexes (in red). Inset: Sedimentation of the complexes after 48 hours. The addition of EDTA to PEI/metal ion complexes results in the elimination of large aggregates, particularly in aqueous solution containing In^{3+}, Ga^{3+}, Ti^{4+}, and Y^{3+} ions

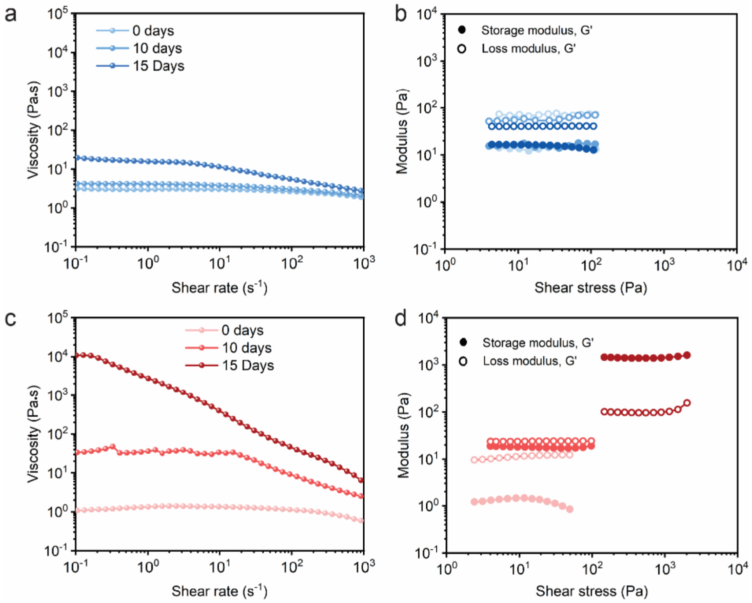

Fig. S5 Viscosity as a function of shear rate for (**a**) PEI/Zn^{2+} and (**c**) PEI/EDTA-Zn^{2+} inks. Storage and loss moduli as a function of shear stress in the linear elastic range for (**b**) PEI/Zn^{2+} and (**d**) PEI/EDTA-Zn^{2+} inks. The rheological properties were characterized for inks stored in ambient environment for 0, 10, and 15 days

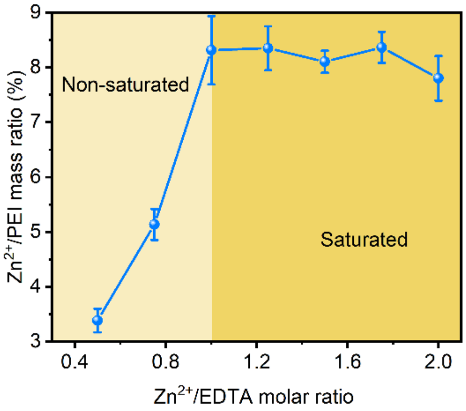

Fig. S6 The content of zinc ions adsorbed on PEI polymers in varying molar ratios of zinc ions to EDTA, measured by ICP-MS analysis of PEI/EDTA-Zn^{2+} inks (pH = 4)

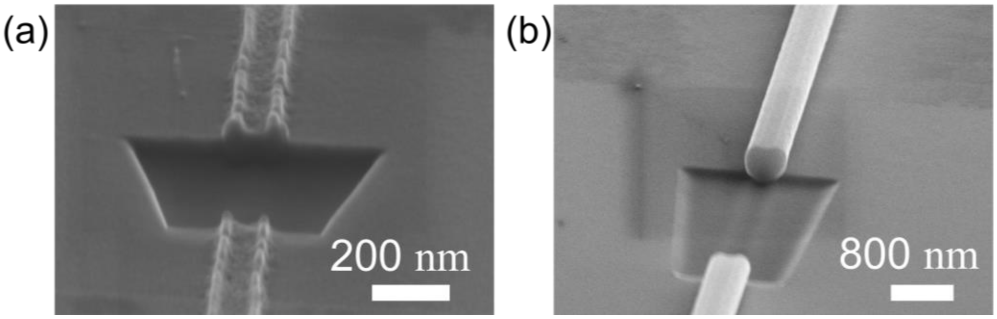

Fig. S7 The cross-sectional morphologies of ZnO rods obtained by sintering 3D-printed polymer precursors with different mass ratios of Zn^{2+} and PEI at 650 °C are shown in (**a**)

3.4 % and (**b**) 8.3 %. When the precursor rod containing unsaturated metal ions is sintered, it collapses into planar and isolated islands consisting of metal oxides. By contrast, when there are saturated metal ions in the precursor, the structure features of sintered products are still visible and remain the same as during ink extrusion.

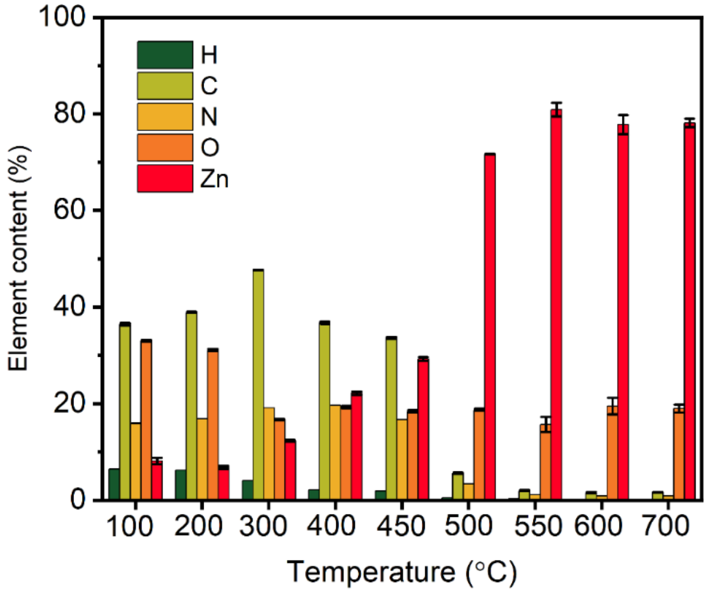

Fig. S8 Element analysis for polymer precursors inks upon sintering to different temperatures for 1 hour

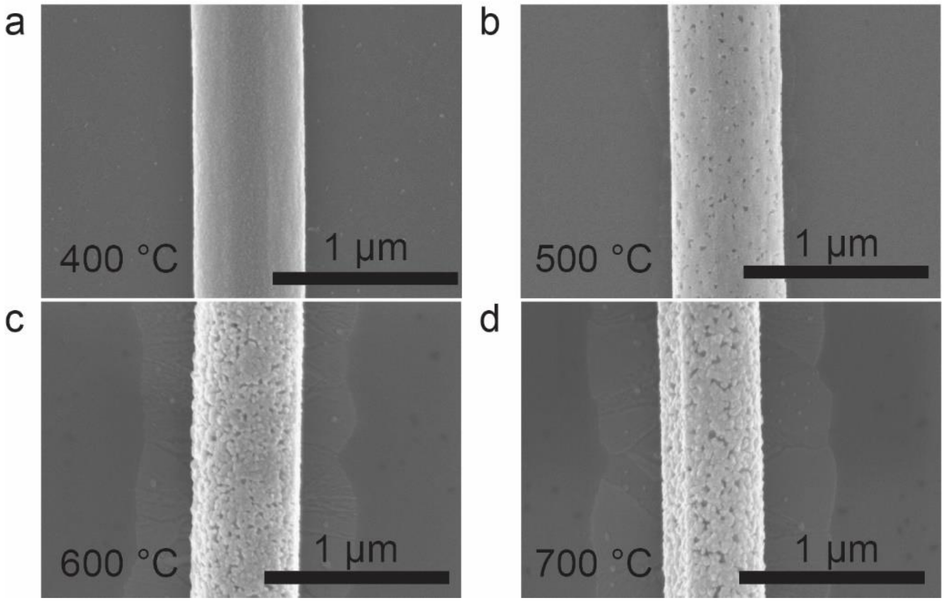

Fig. S9 Surface morphologies of ZnO rods converted from polymer precursor filaments extruded through a 2-μm nozzle by sintering to (**a**) 400 °C, (**b**) 500 °C, (**c**) 600 °C, and (**d**) 700 °C for 1 h in air

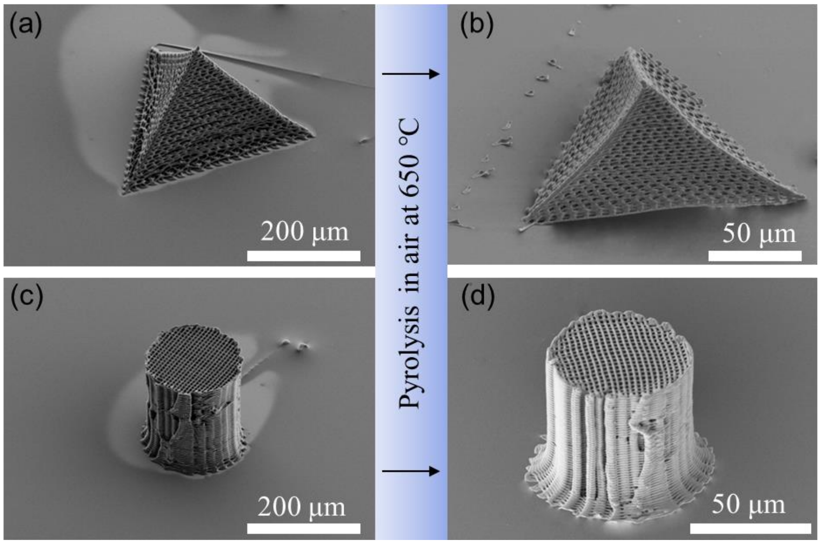

Fig. S10 (**a**) The 100-layer pyramid and (**c**) 150-layer cylinder structures printed using Zn^{2+} ions-containing inks with a 2-μm nozzle underwent an organic-to inorganic transformation upon sintering at 650 °C for 1 hour, forming (**b**) ZnO pyramid and (**d**) ZnO cylinder

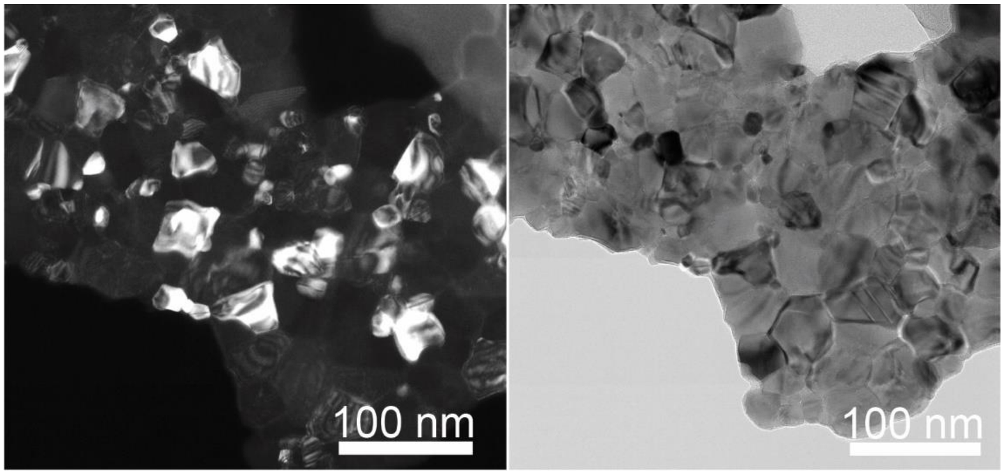

Fig. S11 The bright and dark field TEM images of printed ZnO structures converted from polymer precursor filaments extruded through a 2-μm nozzle by sintering to 650 °C for 1 h in air

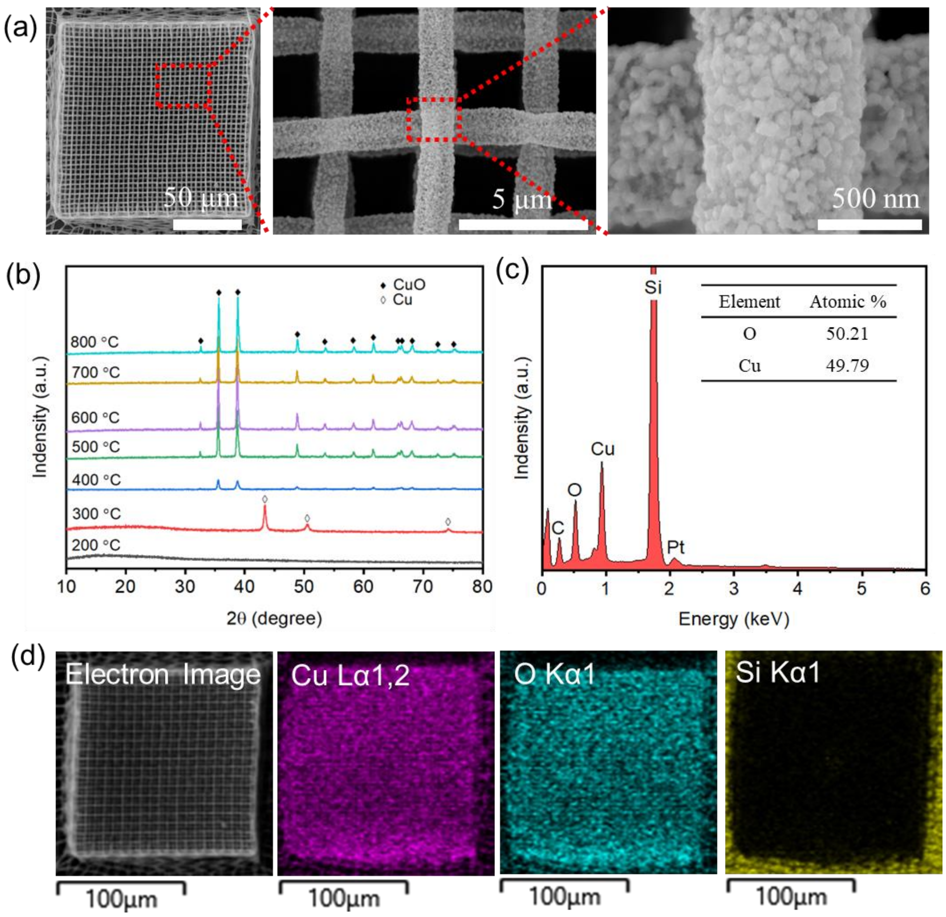

Fig. S12 (**a**) CuO woodpile structure formed by sintering Cu^{2+} ions-containing polymer precursor to 600 °C for 1 hour in air. (**b**) X-ray patterns taken upon sintering the polymer precursor to different temperatures ranging from 200 to 800 °C. The impurity phase i.e., Cu, disappears completely upon heating the printed structures to 400 °C and above. The pyrolyzed products are identified as CuO phase with an onset crystallization temperature of ~400 °C. (**c-d**) The EDS spectrum and element distribution of as-fabricated CuO woodpile shows the atomic percentages of Cu and O consistent with CuO stoichiometry.

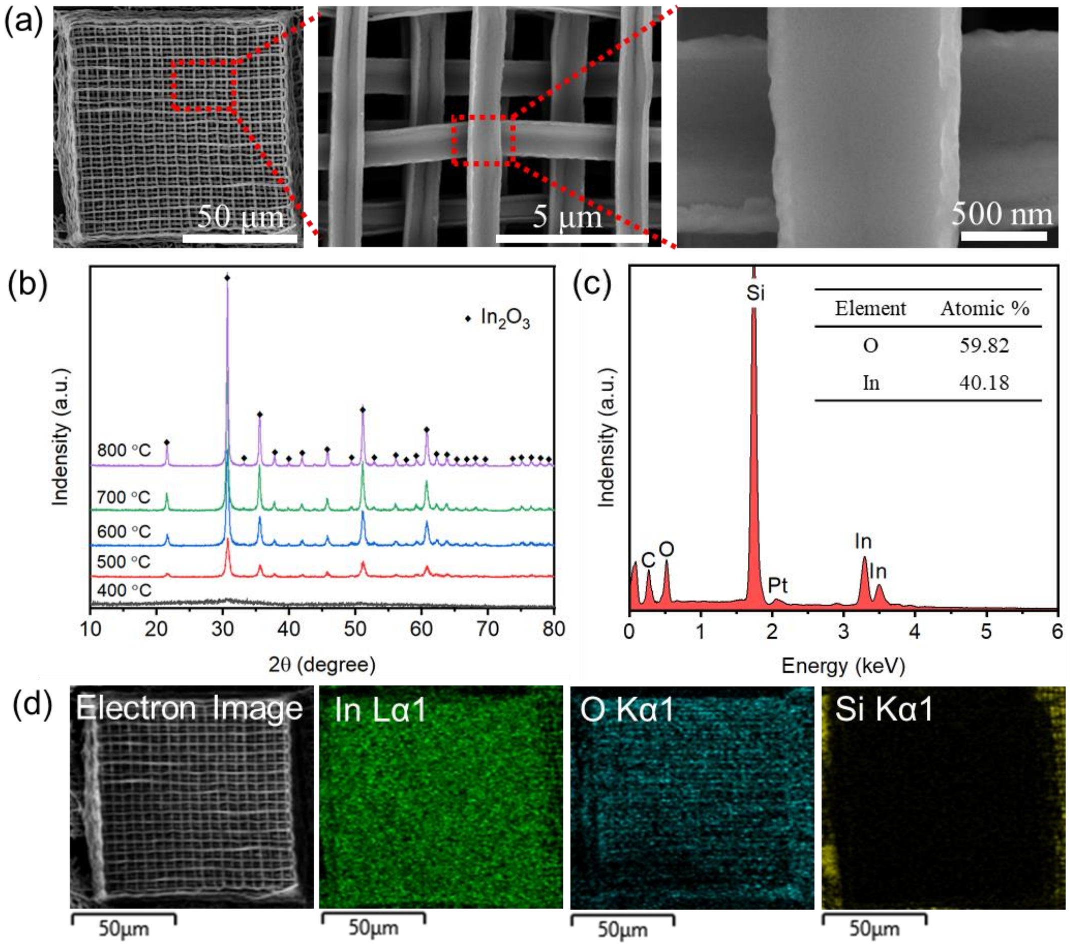

Fig. S13 (**a**) In_2O_3 woodpile structure formed by sintering In^{3+} ions-containing polymer precursor to 600 °C for 1 hour in air. (**b**) X-ray patterns taken upon sintering the polymer precursor to different temperatures ranging from 400 to 800 °C. The pyrolyzed products are identified as In_2O_3 phase with an onset crystallization temperature of ~500 °C (**c-d**) The EDS spectrum and element distribution of as-fabricated In_2O_3 woodpile shows that the atomic percentages of In and O are consistent with In_2O_3 stoichiometry

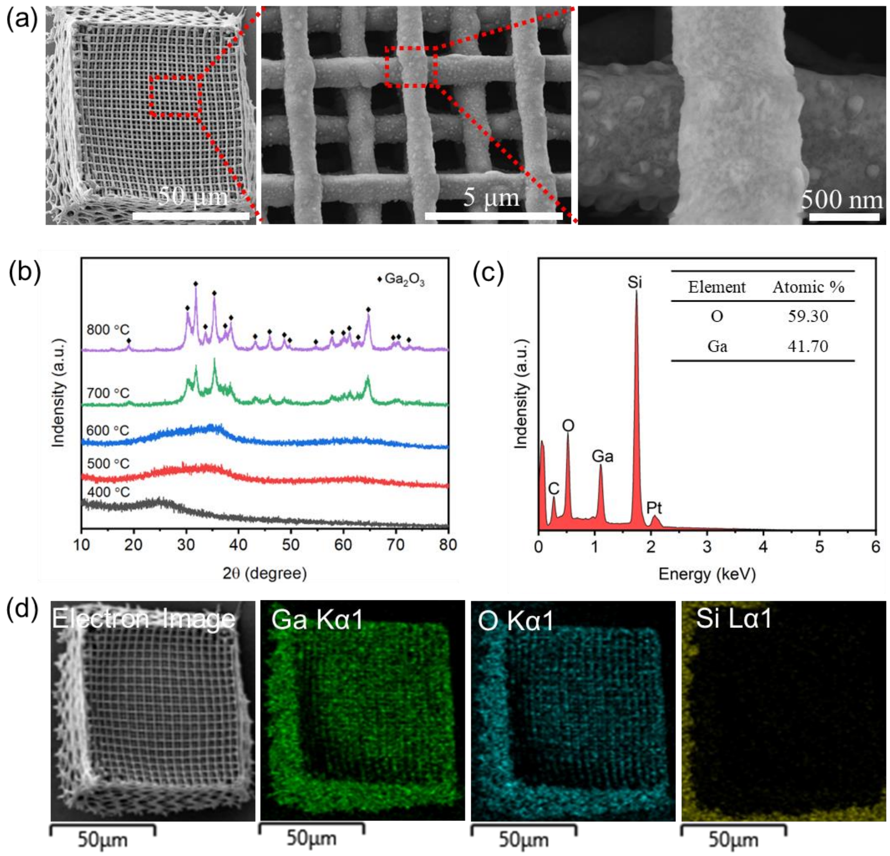

Fig. S14 (**a**) Ga_2O_3 woodpile structure formed by sintering Ga^{3+} ions-containing polymer precursor to 700 °C for 1 hour in air. (**b**) X-ray patterns taken upon sintering the polymer precursor to different temperatures ranging from 400 to 800 °C. The pyrolyzed products are identified as Ga_2O_3 phase with an onset crystallization temperature of ~700 °C. (**c-d**) The EDS spectrum and element distribution of as-fabricated Ga_2O_3 woodpile shows that the atomic percentages of Ga and O are consistent with Ga_2O_3 stoichiometry

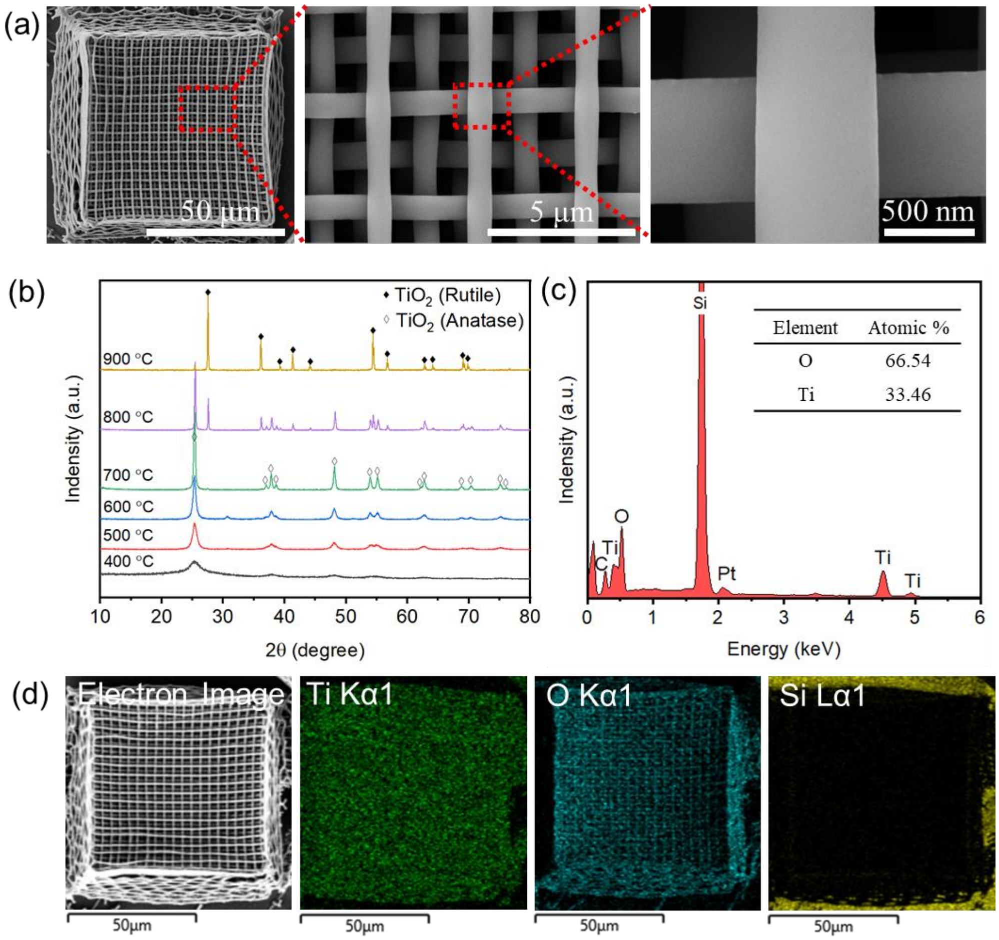

Fig. S15 (**a**) TiO_2 woodpile structure formed by sintering Ti^{4+} ions-containing polymer precursor to 600 °C for 1 hour in air. (**b**) X-ray patterns taken upon sintering the polymer precursor to different temperatures ranging from 400 to 900 °C. The pyrolyzed products are identified as TiO_2 anatase phase with an onset crystallization temperature of ~600 °C. (**c-d**) The EDS spectrum and element distribution of as-fabricated TiO_2 woodpile shows that the atomic percentages of Ti and O are consistent with TiO_2 stoichiometry

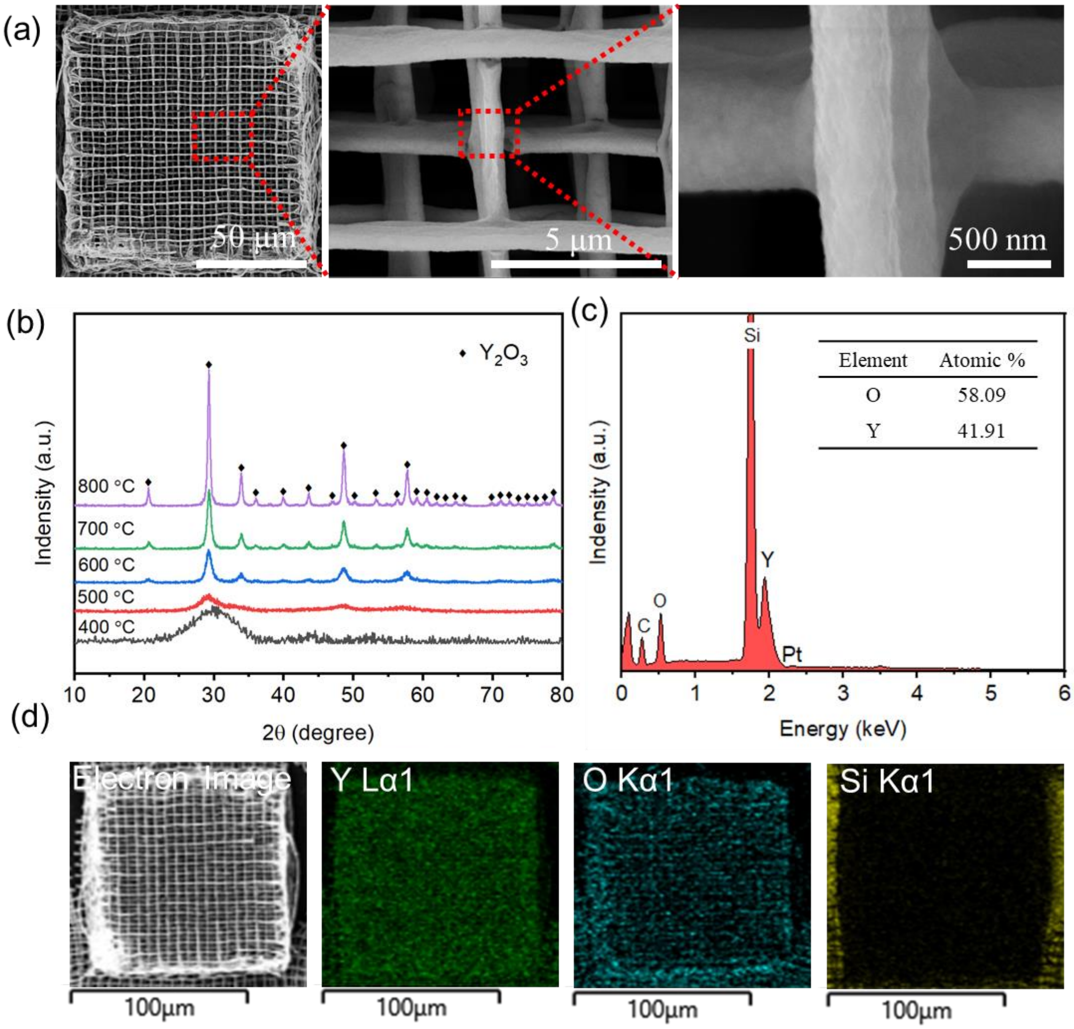

Fig. S16 (**a**) Y_2O_3 woodpile structure formed by sintering Y^{3+} ions-containing polymer precursor to 600 °C for 1 hour in air. (**b**) X-ray patterns taken upon sintering the polymer precursor to different temperatures ranging from 400 to 800 °C. The pyrolyzed products are identified as Y_2O_3 phase with an onset crystallization temperature of ~600 °C. (**c-d**) The EDS spectrum and element distribution of as-fabricated Y_2O_3 woodpile shows that the atomic percentages of Y and O are consistent with Y_2O_3 stoichiometry

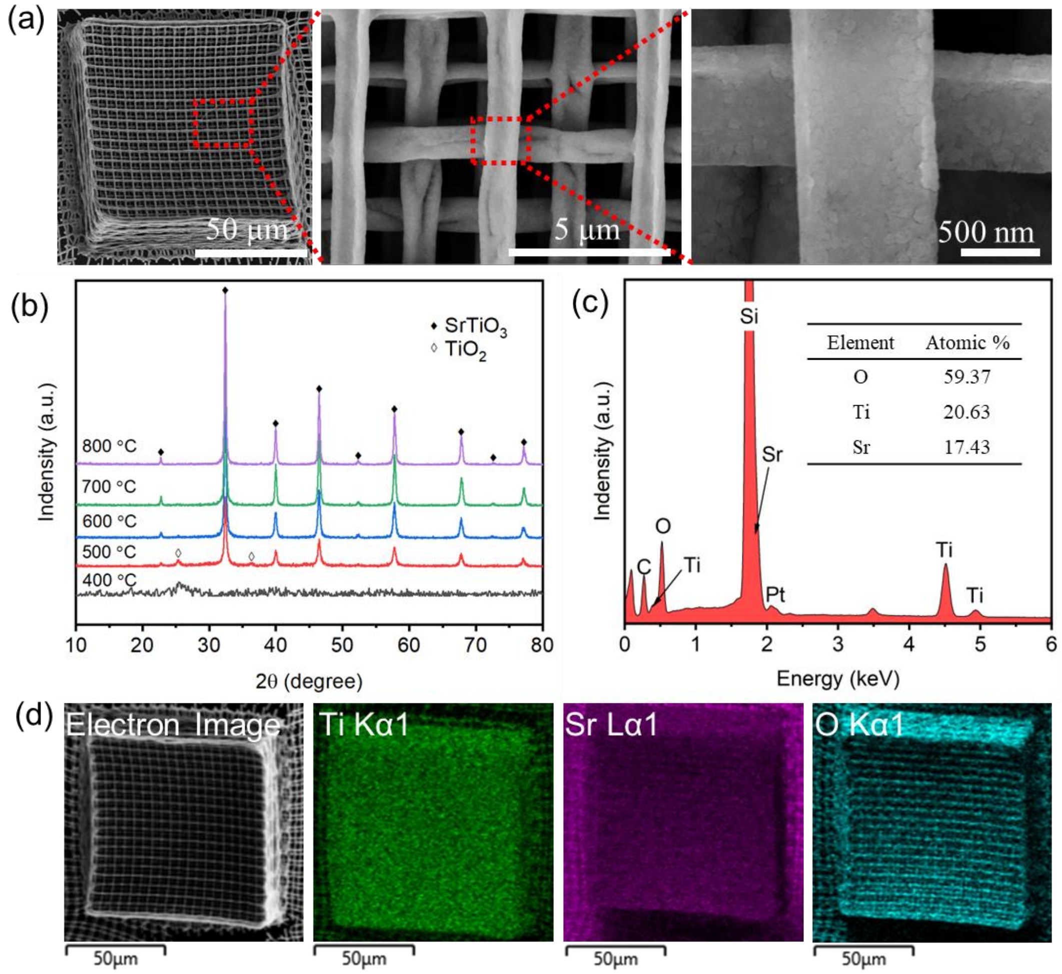

Fig. S17 (**a**) $SrTiO_3$ woodpile structure formed by sintering Sr^{2+} and Ti^{4+} ions-containing polymer precursor to 600 °C for 1 hour in air. (**b**) X-ray patterns taken upon sintering the polymer precursor to different temperatures ranging from 400 to 800 °C. The impurity phase i.e., TiO_2, disappears completely upon heating to 400 °C and above. The pyrolyzed products are identified as $SrTiO_3$ phase with an onset crystallization temperature of ~500 °C. (**c-d**) The EDS spectrum and element distribution of as-fabricated $SrTiO_3$ woodpile shows that the atomic percentages of Sr, Ti, and O are consistent with $SrTiO_3$ stoichiometry

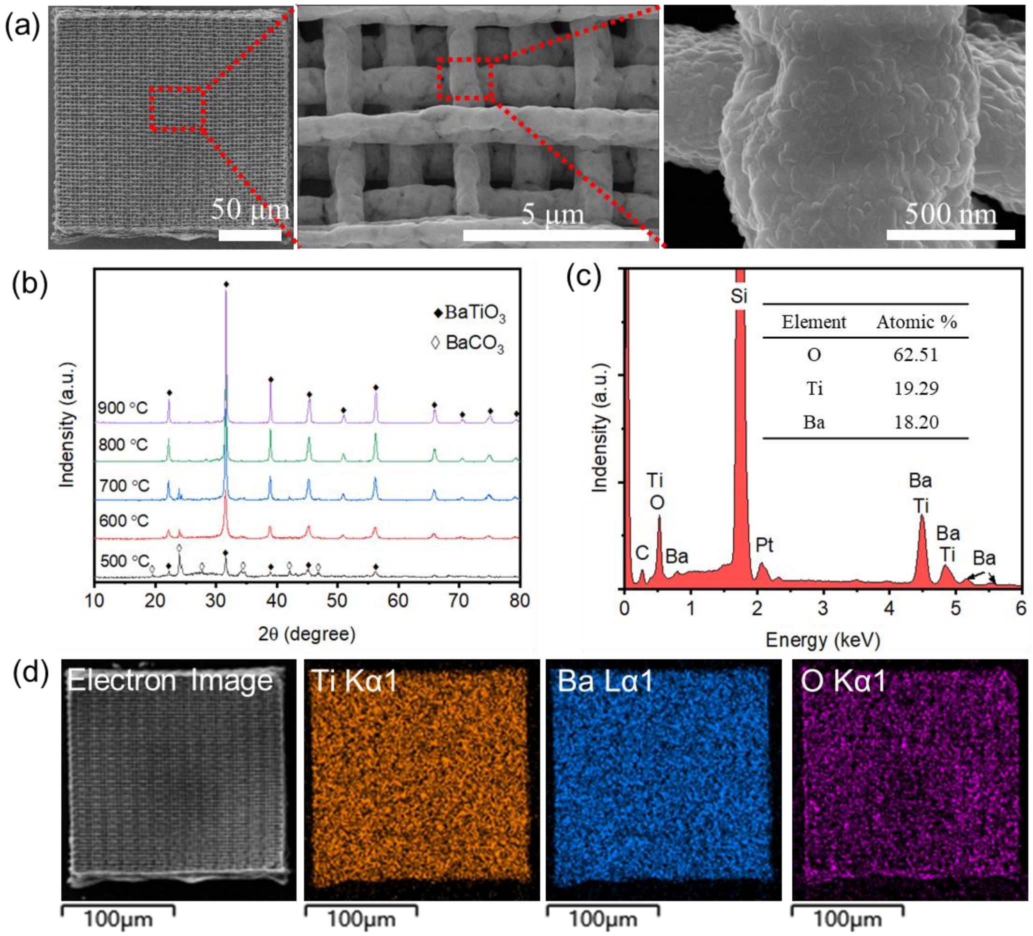

Fig. S18 (**a**) $BaTiO_3$ woodpile structure formed by sintering Ba^{2+} and Ti^{4+} ions-containing polymer precursor to 800 °C for 1 hour in air. (**b**) X-ray patterns taken upon sintering the polymer precursor to different temperatures ranging from 500 to 900 °C. The impurity phase i.e., $BaCO_3$, disappears completely upon heating to 400 °C and above. The pyrolyzed products are identified as $BaTiO_3$ phase with an onset crystallization temperature of ~500 °C. (**c-d**) The EDS spectrum and element distribution of as-fabricated $BaTiO_3$ woodpile shows that the atomic percentages of Ba, Ti, and O are consistent with $BaTiO_3$ stoichiometry

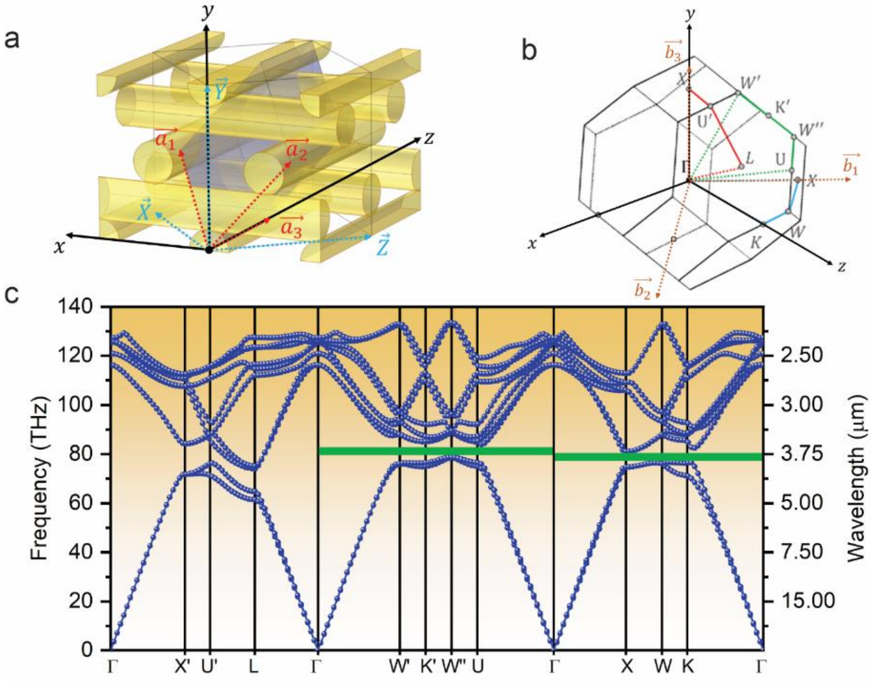

Fig. S19 (**a**) A schematic of a woodpile structure built in x-y-z Cartesian coordinate system, where the $\overrightarrow{a_1}$, $\overrightarrow{a_2}$ and $\overrightarrow{a_3}$ are primitive lattice vectors. $\vec{X}$, $\vec{Y}$ and $\vec{Z}$ are conventional lattice vectors. (**b**) The first Brillouin zone of the FCT Bravais lattice and the scanning path of wavevector in band structure simulation. $\overrightarrow{b_1}$, $\overrightarrow{b_2}$ and $\overrightarrow{b_3}$ are conventional reciprocal lattice vectors. (**c**) Calculated photonic band structure of the 3D-printed ZnO woodpile structure with FCT lattice symmetry

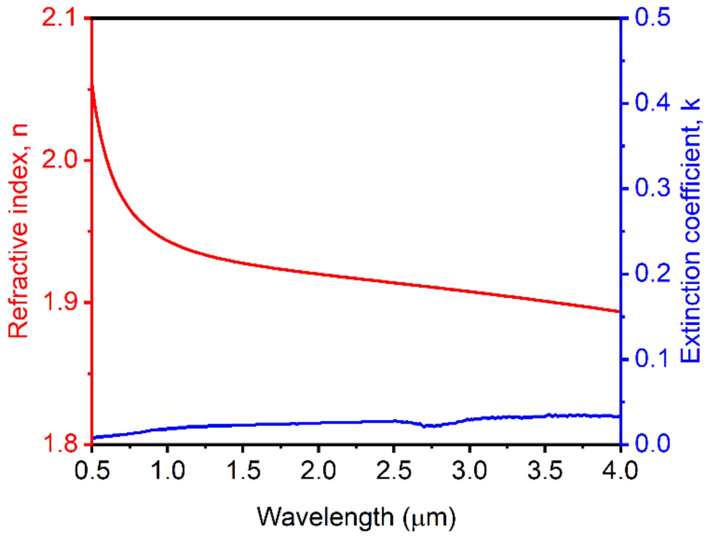

Fig. S20 Refractive index and extinction coefficient of zinc oxide as a function of wavelength [S1, S2]

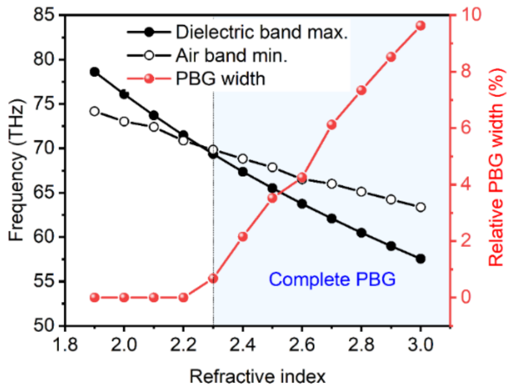

Fig. S21 Band edges (i.e., the maximum value in dielectric band edge and the minimum value in air band edge) and relative photonic band gap (PBG) width as function of constituent material refractive index. When the refractive index increases to 2.3, the complete PBG appears in the FCT woodpile (a = 2.151 μm, d = 0.766 μm, h = 2.961 μm)

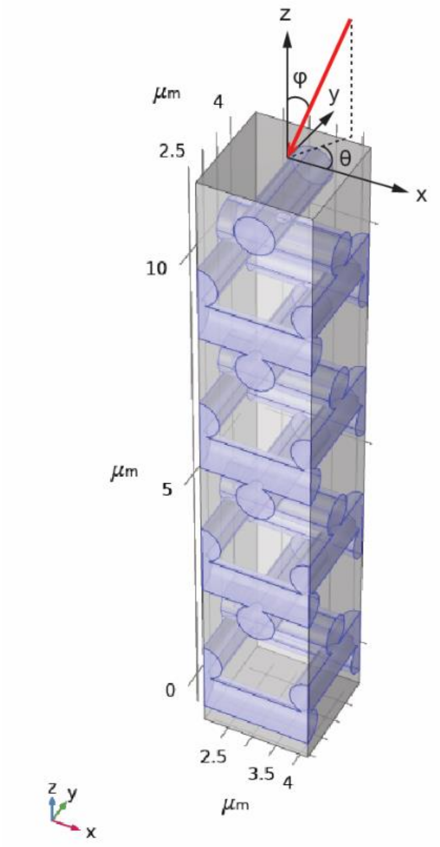

Fig. S22 Illustration for the simulated structure with a finite length of four unit cell in the z direction and the propagation direction of incident light (red line)

Table S1 Wavenumbers values for main FTIR peaks and the corresponding functional groups and their vibration types [S3-S5]

Wavenumber (cm^{-1})	Types of vibration
3278	-OH, $-NH_2$, -NH stretching
2909	$-CH_3$, $-CH_2$, -CH stretching
2832	$-CH_3$, $-CH_2$, -CH stretching
1645	-C=N stretching, $-NH_2$ bending
1576	-NH bending
1466	$-CH_2$ bending
1347	-CH bending
1305	-C-N stretching
1053	-C-N, -C-O stretching

Table S2 The coordinates and k-paths of symmetry points in Brillouin zone of face-centered tetragonal (FCT) Bravais lattice

Symmetry point	z	x	y	K-path
Γ	0	0	0	0
X’	0	0	$\frac{2\pi}{h}$	2.12198
U’	$\frac{\pi}{2a}$	0	$\frac{2\pi}{h}$	2.85224
L	$\frac{\pi}{a}$	0	$\frac{\pi}{h}$	4.14026
Γ	0	0	0	5.94549
W’	$\frac{\pi}{2a}$	$-\frac{\pi}{2a}$	$\frac{2\pi}{h}$	8.30544
K’	$\frac{3\pi}{4a}$	$-\frac{3\pi}{4a}$	$\frac{3\pi}{2h}$	9.04575
W’’	$\frac{\pi}{a}$	$-\frac{\pi}{a}$	$\frac{\pi}{h}$	9.78607
U	$\frac{5\pi}{4a}$	$-\frac{3\pi}{4a}$	$\frac{\pi}{2h}$	10.5264
Γ	0	0	0	12.7205
X	$\frac{\pi}{a}$	$-\frac{\pi}{a}$	0	14.7860
W	$\frac{3\pi}{2a}$	$-\frac{\pi}{2a}$	0	15.8188
K	$\frac{3\pi}{2a}$	0	0	16.5491
Γ	0	0	0	18.7398

Table S3 Partial photonic bandgap edges on the incident angles (Elevation angle, φ) of 16 and 35°, obtained from the woodpile band diagram projected along the ΓX' orientation in different azimuth angles of θ=0, 15, 26.56, 30 and 45°

Azimuth angle, θ (degree)	Elevation angle, φ (degree)	Frequency (THz)	Wavelength (μm)
0	16	85.67076	3.5018
		**72.48027**	**4.1391**
	35	**86.40972**	**3.4718**
		75.32528	3.9827
15	16	85.79369	3.4968
		72.86180	4.1174
	35	83.35510	3.5991
		77.03695	3.8942
26.56	16	85.98194	3.4891
		73.32095	4.0916
	35	79.31826	3.7822
		75.02389	3.9987
30	16	85.85950	3.4941
		73.22729	4.0968
	35	80.48253	3.7275
		76.21041	3.9365
45	16	85.71924	3.4998
		73.35764	4.0896
	35	79.44619	3.7761
		75.31335	3.9834

Supplementary References

[S1] W. L. Bond. Measurement of the refractive indices of several crystals. Journal of Applied Physics **36**(5), 1674-1677 (1965). https://doi.org/10.1063/1.1703106

[S2] M. R. Querry. PhD Thesis, University Missouri, 1985.

[S3] W. Li, J. Wu, S. S. Lee, J. D. Fortner. Surface tunable magnetic nano-sorbents for carbon dioxide sorption and separation. Chemical Engineering Journal **313**, 1160-1167 (2017). https://doi.org/10.1016/j.cej.2016.11.013

[S4] N. Khanam, C. Mikoryak, R. K. Draper, K. J. Balkus. Electrospun linear polyethyleneimine scaffolds for cell growth. Acta Biomaterialia **3**(6), 1050-1059 (2007). https://doi.org/10.1016/j.actbio.2007.06.005

[S5] X. Zhang, X. Zhang, B. Yang, Y. Yang, Q. Chen, Y. Wei. Biocompatible fluorescent organic nanoparticles derived from glucose and polyethylenimine. Colloids and Surfaces B: Biointerfaces **123**, 747-752 (2014). https://doi.org/10.1016/j.colsurfb.2014.10.027
